# Design and Validation of a High-Fidelity Left Atrial Cardiac Simulator for the Study and Advancement of Left Atrial Appendage Occlusion

**DOI:** 10.1007/s13239-025-00773-2

**Published:** 2025-01-27

**Authors:** Keegan Mendez, Manisha Singh, Patrick Willoughby, Beatrice Ncho, Aileen Liao, Susan Su, Megan Lim, Elijah Lee, Mohamad Alkhouli, Hasan Alarouri, Ellen T. Roche

**Affiliations:** 1 Institute for Medical Engineering and Science, Massachusetts Institute of Technology, MA Cambridge, USA; 2 Harvard-MIT Program in Health Sciences and Technology, Massachusetts Institute of Technology, MA Cambridge, USA; 3 Department of Mechanical Engineering, Massachusetts Institute of Technology, MA Cambridge, USA; 4 Boston Scientific Corporation, MN Maple Grove, USA; 5 Department of Cardiovascular Diseases, Mayo College of Medicine, MN Rochester, USA

**Keywords:** LAA, LAAO, Cardiac simulator, Circulatory flow loop

## Abstract

**Purpose:**

Atrial fibrillation (AF) is the most common chronic cardiac arrhythmia that increases the risk of stroke, primarily due to thrombus formation in the left atrial appendage (LAA). Left atrial appendage occlusion (LAAO) devices offer an alternative to oral anticoagulation for stroke prevention. However, the complex and variable anatomy of the LAA presents significant challenges to device design and deployment. Current benchtop models fail to replicate both anatomical variability and physiological hemodynamics, limiting their utility. This study introduces a novel left atrial cardiac simulator that incorporates patient-derived LAA models within a benchtop circulatory flow loop, enabling high-fidelity LAAO device testing and development.

**Methods:**

A rigid, patient-derived left atrium (LA) model was 3D printed from segmented MRI data and modified to accommodate attachment of patient-specific LAA models. A library of LAA geometries was fabricated using silicone casting techniques to replicate the mechanical properties of native tissue. The LA-LAA model was integrated into a circulatory flow loop equipped with a pulsatile pump, pressure sensors, and flow probes, allowing real-time hemodynamic analysis. System tunability was demonstrated by varying heart rate, stroke volume, resistance, and compliance to simulate physiological and pathological conditions.

**Results:**

The simulator accurately replicated LA pressure and flow waveforms, closely approximating physiological conditions. Changes in heart rate, stroke volume, and compliance effectively modulated LAP and LA inflow before and after LAAO. Distinct pressure and flow waveforms were observed with different LAA geometries. Hemodynamic analysis revealed increased left atrial pulse pressure after occlusion, with the greatest increase occurring after complete exclusion of the LAA. The simulator facilitated the evaluation of LAAO device performance, including metrics such as seal and PDL, and served as an effective training tool for iterative device deployment and recapture with visual and imaging-guided feedback.

**Conclusions:**

The left atrial cardiac simulator offers a highly tunable and realistic platform for testing and developing LAAO devices. It also serves as an effective procedural training tool, allowing for the simulation of patient-specific anatomical and hemodynamic conditions. By enabling these advanced simulations, the simulator enhances pre-procedural planning, device sizing, and placement. This innovation represents a significant step toward advancing personalized medicine in atrial fibrillation management and improving LAAO outcomes.

**Supplementary Information:**

The online version contains supplementary material available at 10.1007/s13239-025-00773-2.

## Introduction

**Fig. 1:**
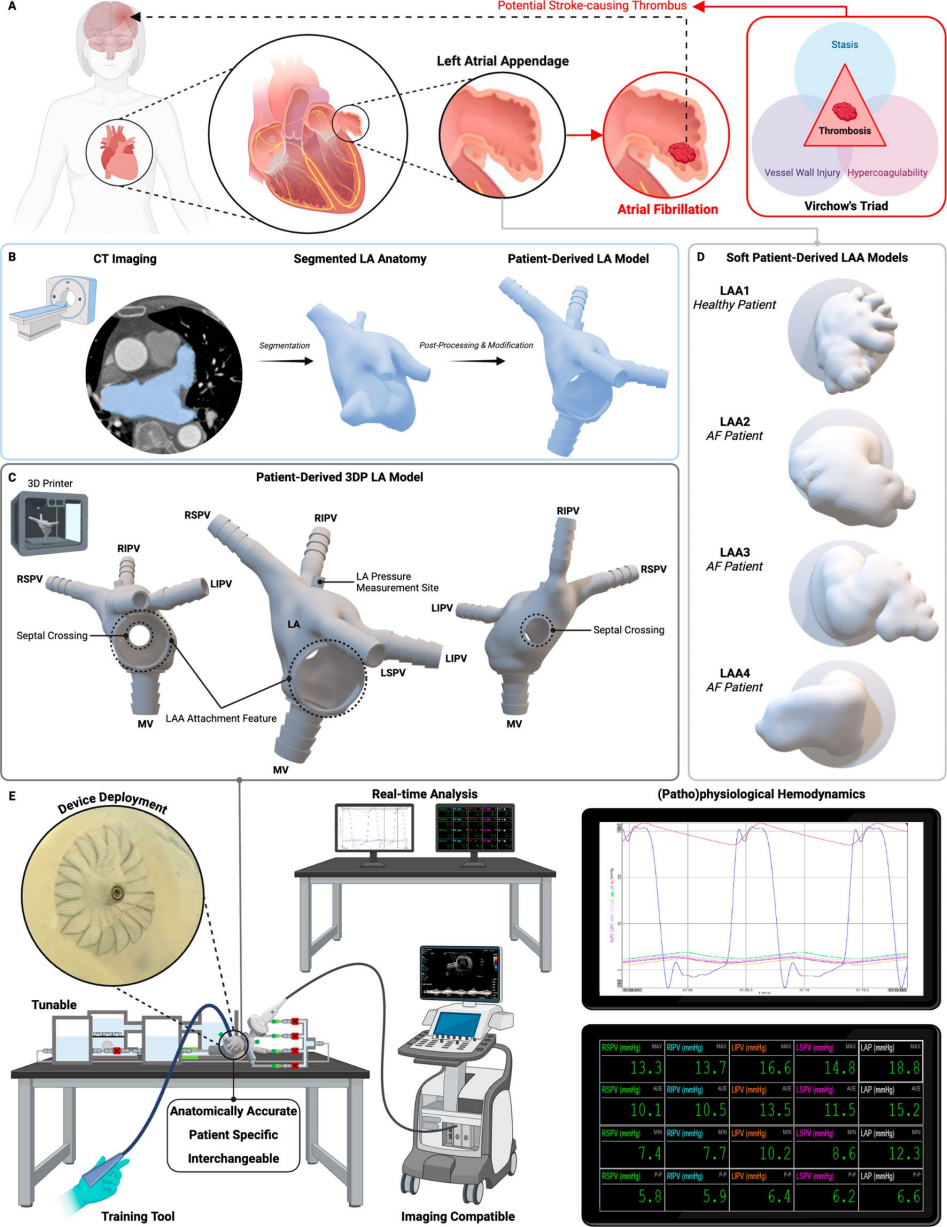
Left atrial cardiac simulator for the study and advancement of left atrial appendage occlusion. **(A)** Location of the LAA within the heart and representation of thrombus formation during atrial fibrillation. AF is caused by abnormal electrical activity. Disordered electrical propagation causes disorganized stimulation of the myocardium and subsequent arrhythmic contractions. AF decreases contractility, resulting in blood stasis and diminished peak flow velocities. AF is also associated with endothelial damage, fibrosis, and inflammation, especially within the LAA, which leads to a prothrombotic and hypercoagulable state. This association is consistent with “Virchow’s Triad”, which synthesizes the pathogenesis of clot formation in the LAA in patients with AF: abnormal blood flow (stasis), endocardial dysfunction (vessel wall injury), and altered hemostasis (hypercoagulability). (**B-C)** Workflow from clinical imaging to rigid, patient-derived LA model. **(B)** Image segmentation, post-processing, and addition of model features for LAA attachment, septal crossing and integration with circulatory flow loop. **(C)** 3D printed rigid patient-derived model. LA: left atrium; LAA: left atrial appendage; RSPV: right superior pulmonary vein; RIPV: right inferior pulmonary vein; LIPV: left inferior pulmonary vein; LSPV: left superior pulmonary vein; MV: mitral valve. **(D)** Library of soft, patient-derived LAA models. *Healthy* indicates LAA from healthy patient. *AF* indicates LAA from patient with atrial fibrillation. AF: atrial fibrillation. **(E)** Left atrial cardiac simulator that can be used to better understand and advance the field of LAAO. The simulator includes a LA model that is anatomically accurate and can accommodate a diverse array of patient specific, interchangeable LAA geometries. The system enables real-time analysis of hemodynamics that can be tuned to mimic physiological and pathological conditions. The simulator is compatible with multiple clinical imaging modalities and allows for repeatable device deployment with visual feedback and measurable hemodynamic changes, making it a highly useful tool for device development and user training

Atrial fibrillation (AF) is the most common clinically significant cardiac arrhythmia, affecting over 33.5 million people globally [1, 2]. Patients with AF face a five-fold increased risk of stroke, primarily caused by embolism of cardiac thrombi originating from the left atrial appendage (LAA) [3, 4]. The LAA, a tubular, blind-ended structure extending from the left atrium (LA), is anatomically complex and exhibits significant inter-patient variability in size, morphology, and internal structure [5–7]. In patients with nonvalvular atrial fibrillation (NVAF), the LAA is the source of 90% of stroke-causing thrombi [8]. The loss of atrial contraction in NVAF promotes blood stasis within the LAA, increasing the risk of thrombus formation and subsequent embolization to the brain (Fig. 1A).

Oral anticoagulation (OAC) is the treatment of choice for stroke prevention in NVAF. However, long-term OAC is associated with significant limitations, including a five-fold increased risk of intracranial hemorrhage, which is associated with mortality rates exceeding 50% in most studies [9–11]. For patients unable or unwilling to tolerate OAC, particularly those at elevated bleeding risk, non-pharmacological approaches have emerged as effective alternatives. These include surgical excision or exclusion and minimally invasive left atrial appendage occlusion (LAAO) via a transcatheter approach [12–15]. LAAO involves mechanically isolating the LAA from systemic circulation with an implanted device and has proven effective in reducing stroke and bleeding risks associated with OAC [16]. Despite these benefits, the majority of LAAO devices remain constrained by rigid, standardized designs, that often fail to accommodate the diverse and complex anatomy of the LAA (Supplementary Fig. 1; Supplementary Table 1). The LAA’s substantial anatomical variability, including its range in size, morphology, and interaction with adjacent cardiac structures, complicates device implantation and increases the risk of suboptimal device implantation. Poor device fit is strongly associated with procedural complications such as peri-device leak (PDL) [17, 18], device-related thrombosis (DRT) [19–21], and pericardial effusion, all of which significantly increase morbidity and mortality [19, 22, 23]. Furthermore, the standard cutoff for acceptable leaks (< 5 mm) is arbitrary and has not been supported by simulation nor by in vitro studies [18]. Addressing these limitations is essential for refining device design, reducing complications, and optimizing LAAO for a broader range of patients.

These challenges specifically underscore the need for advanced models and tools to improve outcomes. Advances in structural heart disease (SHD) interventions have been made with computational modeling and 3D printing (3DP), which enable highly patient-specific procedural planning and simulation. Computational methods such as finite element analysis (FEA) and computational fluid dynamics (CFD) have been applied to simulate cardiac dynamics, predict thrombus risk, and guide device sizing and placement [24–26, 29]. However, CFD is limited by its sensitivity to boundary conditions and material assumptions, which can oversimplify modeling. Similarly, 3DP models often lack dynamic fluid flow and physiologically relevant hemodynamics, which can oversimplify the complex, dynamic environment in which devices are deployed. Virtual reality (VR) systems [27] have also emerged as valuable educational tools, providing clinicians with interactive training to familiarize themselves with procedural steps and assist in selecting appropriate device sizes based on patient anatomy and measurements. However, these systems are constrained by the limited number of cases available for simulation and their narrow focus on clinical training applications rather than exploring fundamental research questions related to LAAO, such as quantifying measurable hemodynamic changes following occlusion or evaluating device performance across diverse physiological conditions.

Benchtop circulatory flow loops address some of these limitations by simulating cardiovascular hemodynamics under controlled conditions, providing a platform for testing, procedural planning, and implanter training. However, most existing flow loops focus on valvular or ventricular dynamics and lack dedicated models for the LA and LAA. Fanni et al. recently advanced the field by developing a high-fidelity 3DP simulator specifically for LAAO [28]. Their model accurately replicates anatomical features, closely mimicking the interaction between LA cardiac tissue and clinical instrumentation, enabling highly realistic procedural simulation by trained operators. While their model focuses on optimizing material properties and faithfully replicating the procedural protocol for real patients, there remains a critical need for models that incorporate dynamic fluid flow and tunable hemodynamic conditions that would allow for robust quantitative evaluation of device performance under a range of physiological and pathological conditions.

Building on these advancements, we present a left atrial cardiac simulator that aims to address these gaps by accounting for inter-patient anatomical heterogeneity through a diverse library of soft, patient-specific LAA models that can be interchangeably coupled to a rigid, patient-derived 3DP LA model. Integrated into a benchtop circulatory flow loop, the simulator enables real-time measurement and tuning of pressure and flow waveforms. Our system has utility in device development and testing in precisely controlled (patho)physiological conditions with quantifiable markers of device performance, as well as in procedural training with direct and imaging-guided feedback. This platform has the potential to enhance procedural outcomes, refine device design, and improve clinician training, ultimately advancing the field of LAAO.

## Methods

### Rigid Patient-Derived LA Model

An open-source database of 100 MRI scans from patients with atrial fibrillation (AF) [30] was reviewed to select a single patient geometry for modification. The dataset included 43 paroxysmal AF, 41 persistent AF, and 16 long-standing persistent AF cases, resulting in 100 patient-specific models. All subjects provided informed consent, and the models were irreversibly anonymized to ensure confidentiality before being made publicly accessible through the database. The chosen geometry exhibited characteristic LA dilation, a common morphological feature in patients with chronic AF [31]. Selection criteria included an LA volume greater than 10 mL, representative of patients with AF and cardioembolic stroke [32], and a long-axis dimension exceeding 32 mm to ensure sufficient space for coupling with a library of LAAs of varying sizes. The selected LA geometry was modified using CAD software (Meshmixer) to isolate the LA and remove the existing LAA. A custom connector with a diameter of 34 mm was designed to enable the interchangeable attachment of LAAs with ostium sizes encompassing approximately over 95% of reported LAA anatomies [33]. The connector’s dimensions were selected to accommodate varying ostial diameters while maintaining compatibility with the underlying LA anatomy. To facilitate procedural simulation, a septal crossing window measuring 10 mm in diameter was incorporated into the model at the inferior fossa ovalis. Its position was determined based on input from a clinician, ensuring anatomical relevance and accessibility for device delivery in variable LAA geometries. The window can be sealed with circular silicone sheets of varying thicknesses and durometers or fitted with a self-sealing port to accommodate delivery sheath insertion. Additional modifications included the incorporation of 3/8-inch OD barbed connectors (5218K748, McMaster) at the pulmonary veins and a 3/4-inch OD barbed connector (5218K75, McMaster) at the mitral annulus to enable seamless integration into the benchtop circulatory flow loop. The resulting model was 3D printed on a Connex500 (Stratasys, Eden Prairie, Minnesota) inkjet-based multi-material 3D printer from a polypropylene-like photopolymer resin (RGD450) with a wall thickness of 1.5 mm.

### Soft Patient-Derived LAA Models

Four patient-specific LAA geometries were selected from MRI data to represent a range of anatomical variability and challenging-to-occlude configurations. LAA geometries were isolated using 3D Slicer and refined in Meshmixer, ensuring preservation of the ostial landing zone required for device deployment. The geometries were chosen in collaboration with clinicians and industry partners, incorporating criteria such as ostial dimensions, length, and overall morphology. The final library included geometries spanning 19.2 to 32.8 mm in ostial diameter and 23.5 to 44.8 mm in length. These dimensions reflect clinically relevant variations and were chosen to encompass both typical and anatomically complex cases to support robust device evaluation and procedural simulation. Each LAA geometry was used to create 3DP molds consisting of a positive core and a negative shell, fabricated in SolidWorks. These molds facilitated silicone casting with Ecoflex 00–30 at 2 mm wall thickness, selected for its translucency and rapid fabrication process. Tensile testing guided wall thickness to match the elastic modulus of the native LAA tissue [28]. One geometry was directly 3D printed using a stiffer silicone material at 1.5 mm wall thickness (SIL30, Carbon) to mimic pathological compliance changes observed in patients with AF [34, 35]. LAAs were attached to the rigid LA model using the custom connector and secured with a silicone rubber adhesive (Sil-Poxy, Smooth-On) to ensure a watertight seal.

### Mock Circulatory Flow Loop

The LA model and interchangeable LAAs were integrated into a benchtop circulatory flow loop designed to mimic physiological and pathological hemodynamics. The system included: (1) a pulsatile pump (BDC Laboratories Pulse Duplicator 1100 or Harvard Apparatus pump, used interchangeably) capable of variable stroke volumes and heart rates; (2) an artificial mechanical valve (Masters Series Mechanical Heart Valve, Abbott) in the mitral position; and (3) tunable resistance valves and compliance chambers mimicking pulmonary and systemic vasculature. Differences between pump type may necessitate reconfiguration of the mitral valve, resistance valves, and compliance chambers to account for variations in system pulsatility. Resistance and compliance elements between the LA and mitral valve mitigated non-physiological suction artifacts from the pump. All tubing was Tygon S3^™^ B-44-3, with 3/8-inch ID tubing for the pulmonary veins (6516T27, McMaster) and 3/4-inch ID tubing for the systemic circulation (6516T33, McMaster). A 1:4 straight-flow rectangular manifold (1023N244, McMaster) distributed flow to the four pulmonary veins. Pressure was measured at the pulmonary vein inlets, within the LA, and within the LAA using PendoTech pressure sensors (PRESS-S-000, PendoTech). For pressure measurements within the LA and LAA, the sensors were connected to 5 F umbilical vessel catheters (Cardinal Health) via Luer locks and positioned in the respective chambers to ensure accurate readings. Flow was quantified using inline Transonic flow probes (ME10PXN, Transonic) coupled to a Transonic flow console (T403 with TS410 modules, Transonic). Data were acquired with a multi-channel data acquisition (DAQ) system (DewesoftX or LabChart, depending on setup) and exported for analysis in MATLAB (MathWorks).

### Left Atrial Appendage Occlusion

#### Hemodynamic Changes

To investigate acute hemodynamic changes following left atrial appendage occlusion (LAAO), pressure and flow were measured before and after device deployment using a 24 mm WATCHMAN FLX™ Left Atrial Appendage Closure Device (Boston Scientific). To simulate hemodynamic changes associated with surgical excision, complete occlusion, or device endothelialization – scenarios in which the LAA is entirely removed or isolated from the LA – pressure and flow were measured after placement of an acrylic cap over the ostium or deployment of an impermeable occlusion device (24 mm coated WATCHMAN FLX, Boston Scientific). The impermeable device consisted of a WATCHMAN FLX with a silicone coating on the polyethylene terephthalate (PET) covering to prevent flow through the fabric to mimic device endothelialization. The WATCHMAN device size was selected according to manufacturer guidelines based on LAA dimensions. Devices were deployed manually prior to coupling the LAA to the LA model to ensure optimal positioning for accurate hemodynamic measurements. Changes in LA pulse pressure were used as a quantifiable measure of occlusion, capturing the effect of device placement on LA compliance. Additionally, changes in LA inflow and outflow, expressed as volume per beat, were assessed to quantify the impact of occlusion on LA compliance. It is important to note that these specific pressure and flow metrics are not traditional clinical measurements for LAAO but are employed here as research tools to assess and quantify occlusion in a controlled experimental setting.

#### Device Testing

To investigate the impact of device material and assess the degree of “occlusiveness” and PDL, two identically sized devices were tested: a standard WATCHMAN FLX with a permeable PET fabric cover and a WATCHMAN FLX with an impermeable silicone coating applied to the PET fabric cover (Fig. 6A.i). Following device deployment, green dye was injected into the LAA distal to the device, and dye washout was monitored visually via video capture (iPhone 14). Captured videos were processed frame-by-frame, focusing on a central region of interest (ROI) within the LAA distal to the device. Dye intensity within the ROI was quantified using a color thresholding method in HSV (Hue, Saturation, Value) color space. Intensity values were normalized, with pre-injection baseline intensity set to 0.0 and the maximum intensity following dye injection set to 1.0. The time to achieve a 40% reduction in intensity (color intensity = 0.6) was used as a surrogate marker for occlusion effectiveness. An endoscopic camera (SpyGlass DS Direct Visualization System, Boston Scientific) was employed to visualize the LA surface of the device, providing additional qualitative data on device position and potential PDLs. Devices were deployed manually prior to coupling the LAA to the LA model to ensure optimal placement for accurate measurement of hemodynamic effects. It is important to note that dye clearance time is not a clinical marker of occlusion and would not typically be measured in practice. Rather, it serves as a quantifiable surrogate metric for use in this simulator-based research setting, enabling standardized comparisons of occlusion effectiveness under controlled conditions.

#### Imaging Compatibility

To evaluate imaging compatibility and demonstrate the utility of imaging modalities for assessing occlusion and device placement, WATCHMAN FLX devices (Boston Scientific) were deployed and visualized using three imaging techniques: an endoscopic camera (1,080-P HD endoscopic camera, NIDADE; 30 frames per second), intracardiac echocardiography (ICE; AcuNav Ultrasound Catheter), and standard echocardiography (Philips Epiq CVx Cardiovascular Ultrasound System with XL14-3 and X5-1 transducers). For direct visualization, the endoscopic camera was employed to assess the LA surface of the device, confirming appropriate positioning and detecting PDLs. PDLs were visualized by injecting colored dye behind the device; the dye’s passage through gaps or around the device was directly observed via the endoscopic camera. ICE was utilized to monitor dye injection into the LAA distal to the device. Flow through the permeable fabric was observed as flow jets directed toward the ICE probe positioned within the LA. Standard echocardiography (2D B-mode and PW mode) was used to measure peak velocity at the ostial edge before and after device placement, as well as during deliberate mispositioning. To facilitate optimal ultrasound propagation, the model was submerged in a water bath during echocardiographic image acquisition. The ultrasound probe was placed directly on the model, and images were obtained by a clinician to ensure optimal views for analysis. Deliberate device misplacement, characterized by significant canting of the device, was performed to induce PDL. This approach enabled a clear distinction between well-placed and mispositioned devices. PDL was quantitatively assessed using echocardiography, with data analyzed in Q-Vue 2.2 (Philips), demonstrating the capability of standard echocardiography to provide measurable data in this research context. It is important to note that these imaging modalities were not used in their traditional clinical capacities. Instead, they were adapted in this research setting to evaluate and quantify occlusion, device placement, and PDL under controlled, simulated conditions.

#### Procedural Training

To demonstrate the simulator’s capability for procedural training and simulation, an experienced operator repeatedly deployed and recaptured a WATCHMAN FLX device (Boston Scientific) under direct visualization. Deployment and recapture were performed using the Access System (Access Sheath and Dilator, Boston Scientific) and Delivery System (Delivery Catheter and Closure Device, Boston Scientific). Each deployment was assessed based on the PASS^™^ (Position, Anchor, Seal, Size) Criteria established by Boston Scientific, ensuring adherence to standard clinical protocols.

## Results

The first step in developing our left atrial cardiac simulator was designing and fabricating a rigid, patient-derived left atrial model that could accommodate soft, patient-specific LAAs of varying shapes and sizes (Fig. 1B-D). Using an open-source database [36] of 100 MRI scans from patients with AF, we selected and segmented a single LA for modification. The segmented model was post-processed to include features for LAA attachment, septal crossing, and integration into a circulatory flow loop (Fig. 1C). The LAA attachment feature was designed with a round geometry and a universal diameter to accommodate a wide range of LAA sizes and shapes. Its symmetrical design supports various LAA geometries with differing short and long-axis ostial dimensions and allows for rotation about the central axis, offering flexibility to simulate varying levels of difficulty in device deployment. The septal crossing window was positioned based on clinician input, ensuring it represented a realistic location for transeptal puncture. This window can be sealed with circular silicone sheets of varying thickness or durometer, allowing for customizable simulation of transeptal crossing during procedural training. The final model was then 3D printed, creating the foundation for the left atrial cardiac simulator.

Next, we developed a library of soft, patient-derived LAA models for attachment to the rigid LA model (Fig. 1D, Supplementary Fig. 2). Using the same open-source database, we selected a diverse range of LAAs with varying geometries. After segmentation and post-processing, the LAAs were isolated by making plane cuts through the LA proximal to the ostial opening, ensuring preservation of the landing zone necessary for device deployment (Supplementary Fig. 2A). Custom molds were then created to produce soft silicone castings for each geometry (Supplementary Fig. 2B). These models captured the size and elasticity ranges representative of LAAs in both healthy adults and patients for whom LAAO would have been appropriate (Supplementary Fig. 2C-D). This workflow yielded a comprehensive library of soft appendages, reflecting both normal and pathological LAA anatomies for coupling with the LA model.

**Fig. 2:**
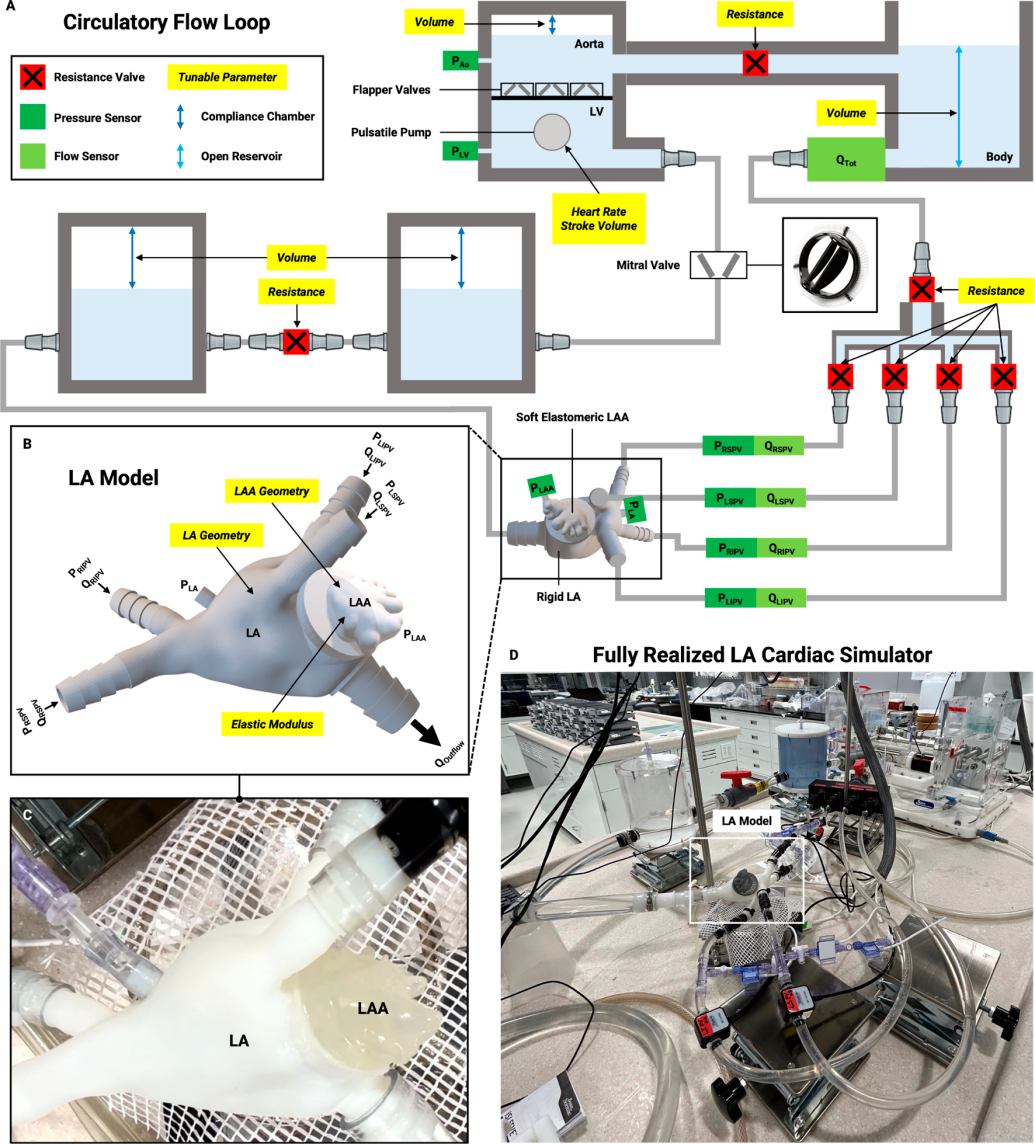
Schematic of circulatory flow loop and incorporation of patient-derived LA model to achieve LA cardiac simulator. **(A)** Schematic of circulatory flow loop with patient-derived LA model, pulsatile pump, commercially available mechanical heart valve in the mitral position, and tunable resistance and Windkessel-based compliance elements to mimic pulmonary and systemic vasculature. Instrumentation and corresponding measured values indicated (Q indicates flow; P indicates pressure). Total flow (Q_Tot_) is measured at the outflow of the pulsatile pump. Flow (Q) is measured at the four pulmonary vein inlets. Pressure (P) is measured at the four pulmonary vein inlets, the left atrium, the left atrial appendage, and the simulated left ventricle and aorta. **(B)** 3D rendering of LA model composed of patient-specific soft elastomeric LAA coupled to 3DP patient-derived rigid LA. The LA model has four inflows (RIPV, RSPV, LSPV, LIPV) and one outflow (MV). The model can be customized by varying the LA geometry (~ days) or interchanging the LAA geometry (~ minutes) from the library of LAA models of varying geometry and material properties. **(C)** Realization of LA model. Complete LA model with LAA1 incorporated into circulatory flow loop. **(D)** Fully realized LA cardiac simulator. RSPV: right superior pulmonary vein; RIPV: right inferior pulmonary vein; LIPV: left inferior pulmonary vein; LSPV: left superior pulmonary vein; LA: left atrium; LAA: left atrial appendage; MV: mitral valve; LV: left ventricular

Following the design and fabrication of our left atrial model and the library of LAA models capturing intra-patient heterogeneity, we integrated these components into a mock circulatory flow loop (Fig. 2). The flow loop comprises a pulsatile pump, the custom LA model, a mechanical mitral valve, and tunable resistance and Windkessel-based compliance elements (Fig. 2A). The LA model includes four inflows, representing the pulmonary veins, and a single outflow, representing the mitral valve (Fig. 2B). The system is equipped with pressure sensors and flow probes to measure total LA inflow, as well as pressure at the pulmonary veins, within the LA and LAA, and in the simulated left ventricle (LV) and aorta. Different soft LAA geometries were easily attached to the rigid LA model, with LAA1 depicted in Fig. 2C. The successful integration of these models into the circulatory flow loop allowed us to fully realize the LA cardiac simulator as designed (Fig. 2D).

**Fig. 3:**
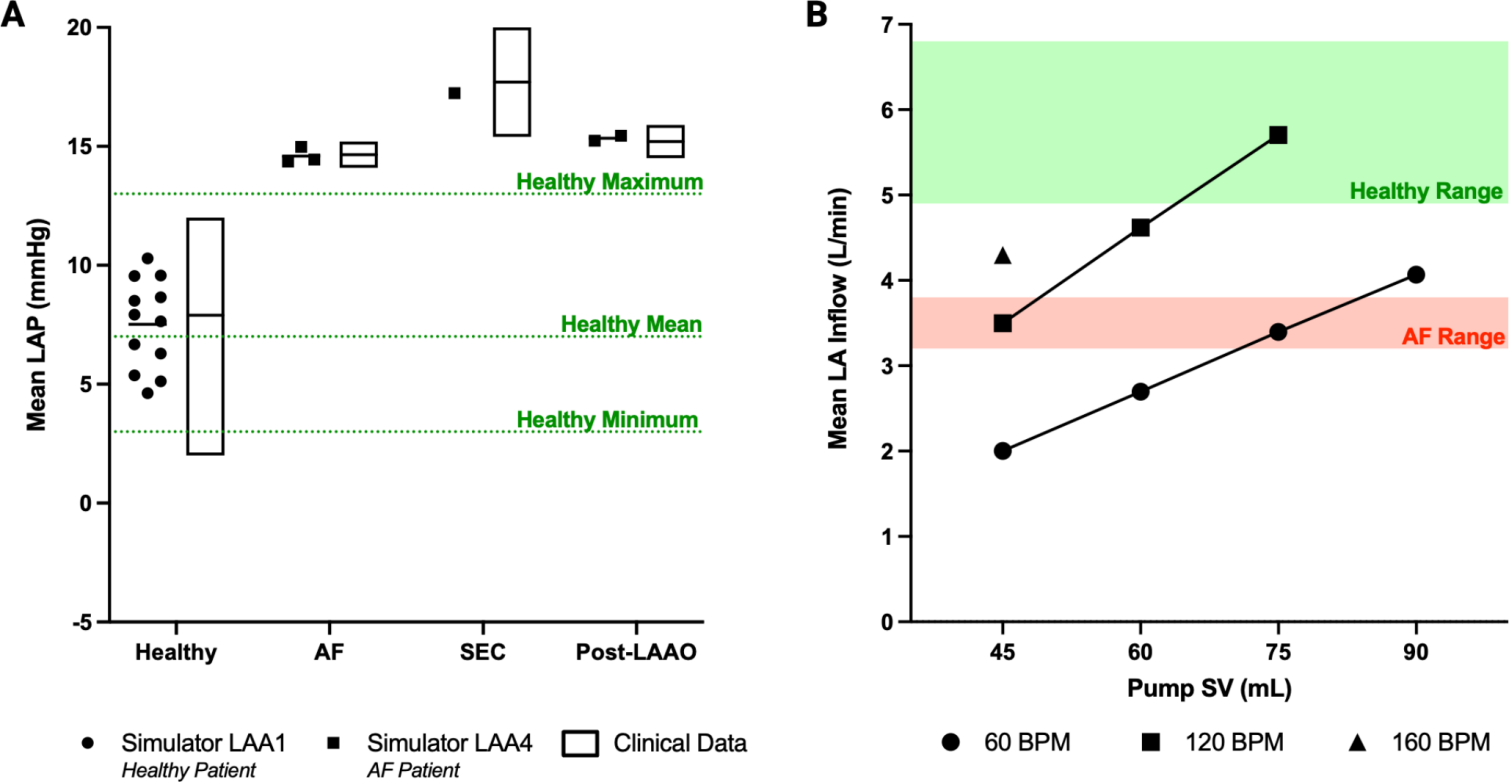
Validation of LA cardiac simulator by demonstrating physiological and pathological hemodynamics. **(A)** Mean LAP achieved in LA cardiac simulator validated against reference data (Supplementary Table 2) from healthy adults (dotted green lines) and clinical data measured in patients with AF (*n* = 435), SEC (*n* = 32) and post-LAAO (*n* = 250). LAA1 (healthy patient) was used in the simulator to collect “Healthy” data. LAA4 (AF patient) was used in the simulator to collect “AF”, “SEC” and “Post-LAAO” data. The simulator was tuned by varying the pump heart rate, pump stroke volume, resistance, compliance and static reservoir height to achieve the target pressure values. Reference data for healthy adults from UpToDate (Supplementary Table 4). Clinical data from collaborators at Mayo Clinic (Supplementary Table 5). LAP: left atrial pressure; AF: atrial fibrillation; SEC: spontaneous echo contrast; post-LAAO: post-left atrial appendage occlusion. **(B)** Mean LA inflow achieved in LA cardiac simulator at varying pump stroke volume (45–90 mL) and pump heart rate (60–160 BPM). Data collected with LAA1. Red shading indicates range of mean LA inflows in patients with AF referenced from the literature (Supplementary Table 3). Green shading indicates range of LA inflows in healthy adults referenced from the literature (Supplementary Table 3). LA: left atrium; SV: stroke volume

To validate our simulator, we demonstrated its ability to replicate both physiological and pathological LA hemodynamics (Fig. 3). First, we validated left atrial pressure (LAP) against reference data from healthy adults [37] (dotted green lines) and clinical data from patients with atrial fibrillation (AF), spontaneous echo contrast (SEC) – an imaging marker associated with increased thrombus formation [38] – and post-LAAO conditions. Benchmark pressure data are provided in Supplementary Table 2 with additional pressure data in Supplementary Tables 4–5. Data collection was performed using LAA1 (healthy patient model) for “Healthy” data and LAA4 (AF patient model) for “AF,” “SEC,” and “Post-LAAO” conditions. By tuning the pump heart rate, pump stroke volume, system resistance, compliance, and static reservoir height, we achieved target pressure values consistent with these conditions (Fig. 3A). The simulator also replicated physiologically relevant left ventricle (LV) and aortic pressure profiles, closely matching idealized Wiggers diagram curves (Supplementary Fig. 3). Next, we validated mean LA inflow against literature-reported data for healthy adults [39–43] and patients with AF [41–43]. By varying pump heart rate and stroke volume, we achieved a range of inflows between 2.0 and 5.7 L/min (Fig. 3B). Benchmark flow data are provided in Supplementary Table 3 with additional flow data in Supplementary Table 6. To simulate AF hemodynamics, we utilized increased pump heart rates (120 BPM) (Supplementary Fig. 4).

A key feature of the simulator is its exquisite tunability and flexibility, allowing for adjustments and modifications to flow loop dynamics and model geometry to achieve different target hemodynamic values. Following the successful validation of physiological and pathological LA hemodynamics, we sought to comprehensively evaluate the complete functionality of the simulator. This included exploring all available pressure and flow measurement points and assessing the impact of pump parameters on LA hemodynamics (Supplementary Figs. 5–6), varying compliance through either Windkessel elements or the material properties of the LAA model, and assessing the impact on LAP (Supplementary Fig. 7) and exploring the effect of different LAA geometries on LA hemodynamics (Supplementary Fig. 8). We could predictably tune LAP, LA inflow, and LA outflow by varying pump HR and pump SV (Supplementary Figs. 5–6). We could decrease compliance and observe a characteristic increase in amplitude of the LAP waveform [34, 35] (i.e., increased LA pulse pressure) (Supplementary Fig. 7). Finally, we noted measurable differences in LA hemodynamics with different LAA geometries (Supplementary Fig. 8), highlighting the importance of using patient-specific models for more rigorous testing and evaluation. However, we believe further study is warranted with additional LAA geometries, along with variation in LA model positioning for the same LAA geometry, to fully explore changes in flow waveforms in response to different LAA geometries.

**Fig. 4:**
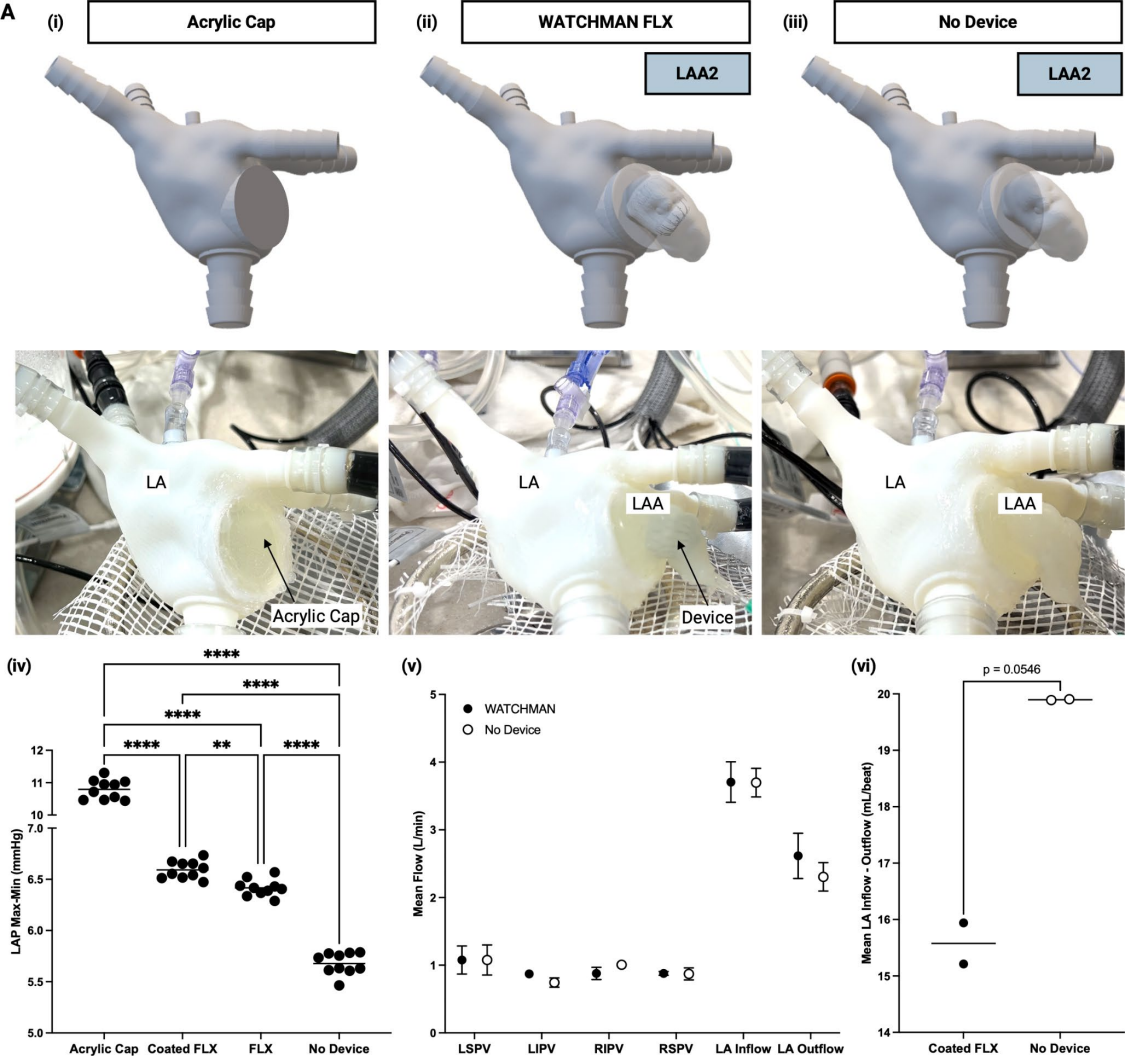
Hemodynamic changes following LAAO in LA cardiac simulator. **(A)** (i) LA model with acrylic cap sealed over ostium to simulate complete LAA exclusion or long-term occlusion following device endothelialization. (ii) LA model with 24 mm WATCHMAN FLX^™^ Left Atrial Appendage Closure Device (Boston Scientific) deployed in LAA2. (ii) LA model with no device in LAA2. (iv) LA pressure amplitude (LAP_PulsePressure_ = LAP_maxima_ – LAP_minima_) before (No Device) and after LAAO with a 24 mm FLX (24 mm FLX) or a silicone coated impermeable 24 mm FLX device (Coated 24 mm FLX), or exclusion with an acrylic cap (Acrylic Cap). All data collected at 60 BPM and pump SV of 90 mL. Dunnett’s T3 multiple comparisons test, **** <0.0001, *** <0.0002, ** <0.0021, * <0.0332, ns = not significant. (v) Mean flow before (No Device) and after LAAO with a silicone-coated impermeable FLX device (Coated FLX). Inflow is defined as the sum of the mean flow through the four pulmonary veins. All data collected at 70 BPM and pump SV of 70 mL or 80 mL. (vi) Mean difference in volume per beat between inflow and outflow before (No Device) and after LAAO with a silicone coated impermeable FLX device (Coated FLX). All data collected at 70 BPM and pump SV of 70 mL or 80 mL. Paired t test, *p* = 0.0546. LAAO: left atrial appendage occlusion; LA: left atrium; LAA: left atrial appendage; SV: stroke volume; PV: pulmonary vein. RSPV: right superior pulmonary vein; RIPV: right inferior pulmonary vein; LIPV: left inferior pulmonary vein; LSPV: left superior pulmonary vein; LA: left atrium; LAA: left atrial appendage; MV: mitral valve; LV: left ventricular

After demonstrating the tunability of the system, we utilized the simulator to investigate hemodynamic changes following LAAO (Fig. 4). We measured LAP under three conditions: complete exclusion of the LAA using an acrylic cap (Fig. 4.i), deployment of a standard WATCHMAN device (24 mm WATCHMAN FLX, Boston Scientific), and deployment of a modified WATCHMAN device with an impermeable silicone coating (not intended for clinical use) to simulate a “healed” device after endothelialization (Fig. 4.ii). These were compared to baseline LAP before occlusion (Fig. 4.iii). Our findings revealed a significant increase in LA pulse pressure following LAAO across all three techniques (Fig. 4.iv). The observed increase in pulse pressure reflects a decrease in compliance (Supplementary Fig. 7), as excision/occlusion reduces the total LA chamber volume and eliminates the LAA as a compliance reservoir. Among the techniques, complete exclusion with the acrylic cap resulted in the highest pulse pressure increase, likely because it entirely sealed the ostium and removed the LAA as a compliance vessel. In contrast, the standard WATCHMAN device, being compliant and permitting residual flow shortly post-implantation primarily through the fabric of the device, caused a smaller increase in pulse pressure. Over time, endothelialization would restrict flow through the standard device, approximating the hemodynamics of the impermeable modified WATCHMAN device. As expected, the modified device, intended to mimic the “healed” device, resulted in higher pulse pressure compared to the standard WATCHMAN, more closely resembling the complete occlusion achieved with the acrylic cap. However, the increase was modest, suggesting residual compliance in the “healed” device/LAA, potential minor leaks around the device, allowing residual LAA compliance function, or that the LAA’s role as a capacitance chamber may be minor. These findings highlight the utility of our simulator for measuring parameters that can be used as indices of occlusion effectiveness, enabling differentiation between levels of occlusion across devices and techniques.

We also measured LA inflow and outflow following LAAO and observed findings indicative of decreased compliance post-occlusion (Fig. 4.v-vi). Specifically, we measured pulmonary venous flow into the LA (LA inflow) and flow immediately distal to the LA (LA outflow) (Fig. 4.v). LA inflow was assumed to be the total sum of flow from all four pulmonary veins. Data were collected both before and after device deployment at two pump settings (70 mL and 80 mL SV at 70 BPM). To assess compliance, we compared the difference between inflow and outflow, expressed as volume per beat (Fig. 4.vi). We hypothesized that a more compliant LA/LAA would result in larger differences between inflow and outflow, as the chamber could accommodate and store more volume. Conversely, a less compliant LA/LAA would yield smaller differences, reflecting a more rigid structure unable to expand with increased flow. As predicted, we observed a decrease in volume per beat after device placement, consistent with reduced compliance. These findings further validate the utility of our simulator for measuring quantifiable markers of occlusion. This capability enables differentiation between occlusion states or pathological conditions that affect LA compliance.

**Fig. 5:**
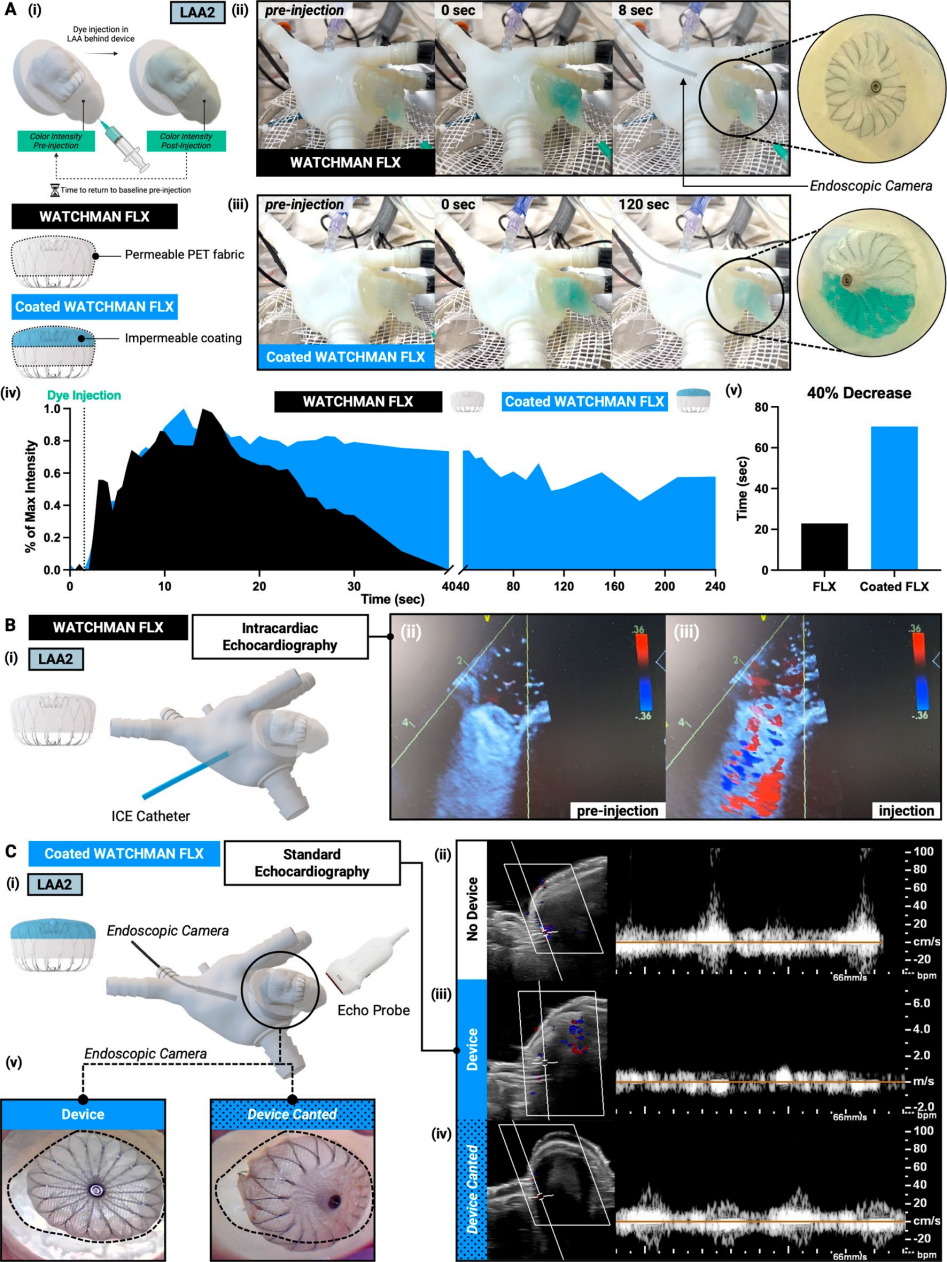
Evaluating LAAO in LA cardiac simulator. **(A)** Comparing occlusiveness of different devices in the cardiac simulator. (i) Following device deployment, a green dye was injected into the LAA distal to the device while color intensity was monitored in the LAA. The time to return to baseline color intensity in the LAA pre-injection was used as a surrogate for the degree of occlusiveness. Two 24 mm WATCHMAN FLX devices were deployed. The first device was a normal WATCHMAN FLX with a permeable polyethylene terephthalate (PET) fabric cover. The second device was a WATCHMAN FLX with an impermeable silicone coating on the PET fabric cover. (ii-iii) Dye was injected into the LAA and could be monitored visually for washout while pressure was measured continuously. (iii-iv, right) An endoscopic camera was used to visualize the LA portion of the device. (iv) Color intensity in the LAA following dye injection. Data are normalized from pre-injection baseline intensity to maximum intensity reached following dye injection. Time above baseline intensity indicated by shaded region. Time of dye injection indicated by vertical dashed line. (v) Time for color intensity in the LAA to decrease by 40% from maximum intensity reached post-injection. (**B)** Using intracardiac echocardiography (ICE) to evaluate device placement and occlusiveness in the cardiac simulator. (i) Schematic of device placement and ICE catheter positioning within the LA. (ii) ICE image pre-injection showing device placement with no flow through the permeable PET fabric cover. (iii) ICE image during injection showing flow from the LAA through the permeable PET fabric cover towards the catheter in the LAA. (**C)** Using standard echocardiography to evaluate device placement and the presence of PDL in the cardiac simulator. (i) Schematic of device placement, echo probe positioning with respect to the LAA, and endoscopic camera positioning within the LA. (ii-iv) Flow as measured by echocardiographic inside the LAA behind the device. (ii) Flow with no device in the LAA. (iii) Flow with the device placed in the LAA. (iv) Flow with the device deliberately misplaced in the LAA. (v) Device (mis)placement can be directly visualized using an endoscopic camera

After demonstrating that the simulator could measure changes in occlusion states, we used it to evaluate specific device performance (Fig. 5). To assess the impact of device material and the degree of “occlusiveness” or PDL – key markers of procedural success – we tested two different devices and monitored dye clearance within the LAA distal to the device (Fig. 5A.i). Dye clearance time, measured as the duration for peak dye intensity in the LAA to return to baseline, served as a quantitative marker for occlusion efficacy. We deployed two identically sized devices, selected per manufacturer guidelines based on LAA dimensions. The first was a standard WATCHMAN FLX with a permeable PET fabric cover, and the second was a modified WATCHMAN FLX with an impermeable silicone coating applied to the PET cover. Following deployment, green dye was injected into the LAA distal to the device, and dye dilution was visually monitored (Fig. 5A.ii-iii). An endoscopic camera captured images of the LA portions of the standard (Fig. 5A.ii, right) and modified devices (Fig. 5A.iii, right). Color intensity in the LAA was measured and normalized from pre-injection baseline intensity to maximum intensity following dye injection (Fig. 5A.iv). For the standard device, peak dye intensity decreased by 40% within 22.9 s and returned to baseline within 40 s, indicating flow through the permeable PET cover (Fig. 5A.v). This reflects the device’s behavior immediately post-deployment, before endothelialization. In contrast, for the coated device, peak dye intensity decreased by 40% over 70.4 s and remained above baseline through 240 s, indicating a higher degree of occlusion (Fig. 5A.v). The impermeable coating prevented flow through the device cover, simulating long-term device behavior after endothelialization. However, the steady dye intensity decline suggests incomplete occlusion, likely due to PDL around the coated device. These results demonstrate the simulator’s capability to evaluate device-specific performance, including occlusiveness and PDL, under both acute and chronic conditions.

After deploying the devices into the simulator, we utilized clinical imaging techniques to visualize the devices and evaluate their performance (Fig. 5B, C). Using intracardiac echocardiography (ICE; Fig. 5B.i), we observed flow through the permeable PET fabric cover toward the ICE probe following dye injection distal to the device (Fig. 5B.ii-iii). Standard echocardiography was also used to assess device placement and measure PDL (Fig. 5C.i). We conducted flow measurements at the LAA ostium wall under three conditions: (1) no device present, measuring baseline flow into and out of the LAA (Fig. 5C.ii), (2) device deployed, measuring a reduction in flow at the same region indicative of successful occlusion (Fig. 5C.iii), and (3) deliberate device misplacement, intentionally canting the device into the LA, resulting in a return of flow at the same region indicative of PDL due to poor positioning (Fig. 5C.iv). An endoscopic camera provided additional visualization of the LA portion of the device, confirming proper placement or deliberate misplacement (Fig. 5C.v). These experiments highlight the simulator’s compatibility with imaging techniques for quantitative assessment of device performance, including occlusion efficacy and potential complications such as PDL from improper device positioning.

**Fig. 6:**
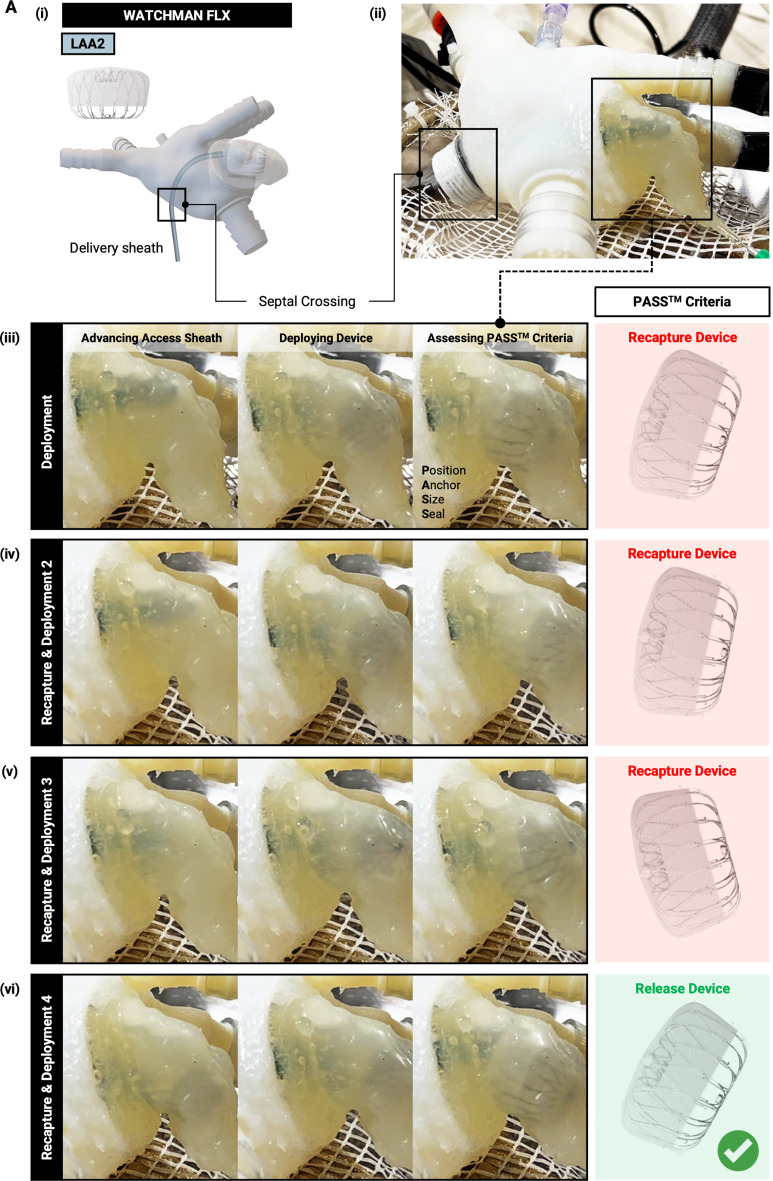
LAAO procedural training in LA cardiac simulator. **(A)** Repeated device deployment and recapture performed by an experienced user using the Access System and Delivery System in the cardiac simulator. (i) Schematic of LA model that allows septal crossing with a delivery sheath and device deployment into the LAA. (ii) LA model with delivery sheath advanced into the LAA. LAA model allows direct visualization for enhanced user-feedback. (iii-vi) Repeated device deployment and recapture until PASS^™^ (position, anchor, size, seal) criteria are met. Device release (vi, right) when user is satisfied with device deployment

Finally, we demonstrated that the simulator could serve as a valuable tool for procedural planning and user training in a realistic, clinically relevant physical environment (Fig. 6). A trained operator utilized the simulator to practice procedural steps, including septal crossing and repeated device deployment and recapture, using the Access System (Access Sheath and Dilator, Boston Scientific) and Delivery System (Delivery Catheter and Closure Device, Boston Scientific) (Fig. 6A.i). The model enabled the operator to practice deploying and repositioning the WATCHMAN FLX device with direct visualization of the device within the LAA (Fig. 6A.ii). The use of clear fluid further allowed intracardiac visualization using an endoscopic camera inserted through one of the pulmonary veins. This setup enabled the operator to repeatedly deploy and recapture the device until achieving satisfactory placement, assessed by the PASS Criteria (Fig. 6A.iii-vi). Each criterion could be evaluated using the following approaches: Position by visual inspection externally or via endoscopy/echocardiography; Anchor by pullback testing and inspection for device movement externally and internally; Seal by dye injection or hemodynamic changes as demonstrated in Figs. 4 and 5 using measurable indices for occlusion and seal; and Size by external measurement of device compression or internal measurement via endoscopy/echocardiography.

## Discussion

Current state-of-the-art cardiac simulators fall short of replicating the intricate anatomical and hemodynamic features of the left atrium and the left atrial appendage. Existing simulators are primarily focused on valvular or ventricular dynamics with applications in valve repair or replacement and ventricular functional assessment. There is a lack of dedicated simulators specifically designed for LAAO that adequately capture the unique structural complexity and hemodynamic physiology of the LA and LAA such that they are suitable for robust research and device testing. To advance the field of LAAO, there is a critical need for simulators that combine anatomical fidelity with physiologically accurate hemodynamics. Such simulators could significantly enhance pre-procedural planning and support the development and evaluation of novel occlusion devices and techniques in a realistic and inclusive environment.

While existing static 3D printed LA models have been associated with improved outcomes, including optimized device selection, reduced procedure times, and lower probabilities of PDL [44–47], these models lack key features critical to LA anatomy and physiology. They cannot replicate physiologically relevant hemodynamics or allow fine-tuning of parameters such as heart rate, pulmonary venous return, and compliance to mimic healthy and pathological states. Additionally, these models are often single prints that do not offer the facile exchange of LAA geometries to capture inter-patient anatomical diversity. Enhancing these models by integrating dynamic flow systems, tunable hemodynamics, and modularity to accommodate various LAA geometries could yield even greater improvements in procedural planning, device development, and clinical training. For example, the ability to test devices across a spectrum of anatomies and physiological conditions could lead to more refined device designs and improved procedural techniques. Ultimately, these enhancements could translate to better patient outcomes, reduced complication rates, and a more comprehensive understanding of LAAO under diverse clinical scenarios.

In this work, we introduce a left atrial cardiac simulator designed to address the limitations of current models. Our simulator integrates a rigid, patient-derived LA model with soft, interchangeable patient-specific LAA geometries. This modular design is incorporated in a circulatory flow loop equipped with a pulsatile pump, a mitral valve, and tunable vascular resistance and compliance. We validated the simulator by replicating left atrial pressure and left atrial inflow conditions representative of both healthy adults and patients with atrial fibrillation. By adjusting parameters such as heart rate, stroke volume, and compliance, we successfully recreated a spectrum of physiologically realistic hemodynamic states, ranging from healthy to pathological. While our maximum achieved mean inflow (5.7 L/min) is below the upper range reported for healthy adults at rest, LA inflow is significantly reduced in AF due to shorter diastolic filling times and the absence of atrial contraction. Thus, our model provides a reasonable approximation of LA inflow conditions in patients with AF who may require LAAO. To simulate AF, we increased the pump heart rate to reflect shorter diastolic filling times. While the resulting dynamics more closely resemble atrial flutter due to the pump’s regular rhythm, future work will focus on more accurately capturing AF-specific dynamics by replicating its characteristic “irregularly irregular” rhythm.

In addition, the simulator was used to evaluate the hemodynamic changes induced by LAAO using various occlusion methods. Quantitative metrics, such as pulse pressure changes and compliance reduction, could be measured, providing valuable data to assess the effectiveness of occlusion techniques and devices. Hemodynamic analysis revealed increased LAP following LAAO, with the greatest increase occurring after complete exclusion of the LAA. These data corroborate recent clinical findings of a small but significant increase in LAP immediately post-LAAO [48]. When evaluating pressure and flow waveforms, it is notable that the LA in our model is not actively contractile; instead, it exhibits passive motion driven by pulsatile flow and inherent elasticity. As a result, the model lacks the characteristic a-wave associated with the “atrial kick,” which occurs at the end of ventricular diastole just before mitral valve closure. While this work focuses on achieving physiologically relevant mean values and ranges, future efforts will aim to further mimic the biomechanics of the LA to more closely replicate realistic waveforms.

Following validation and demonstration that the simulator could be used to collect measurable, quantifiable data relevant for LAAO, we used it to evaluate clinically approved occlusion devices. Device performance was assessed under different conditions, including material properties, device sizing, and positioning. Metrics such as dye clearance time and detection of PDL provided valuable insights into occlusion effectiveness. Importantly, the simulator supported various imaging modalities, enabling visualization of device placement and occlusion outcomes, as well as the quantification of performance metrics like seal efficacy and flow reduction. These findings highlight the potential of our simulator as a valuable tool for device research and development.

Finally, the simulator proved to be a robust procedural training tool, offering clinicians a realistic environment to practice device deployment, repositioning, and recapture. Using direct and imaging-guided feedback, the simulator facilitated the assessment of key procedural criteria, such as the PASS Criteria, enhancing operator learning and confidence. These iterative procedures highlight the simulator’s utility as a training tool, providing clinicians with a realistic environment to practice device manipulation, optimize sizing and positioning, and troubleshoot potential challenges. Importantly, the simulator supports the implantation of multiple device sizes into a single LAA anatomy. Following implantation, percent compression – an essential metric for successful device fit – can be assessed externally through direct measurement or internally via endoscopy/echocardiography. Future improvements could include the integration of sensors into the model’s walls to measure force and test device compression in real-time, further enhancing the simulator’s utility for device evaluation and procedural optimization. Additionally, the simulator offers potential applications in educational settings, including industry-sponsored workshops and conference demonstrations, where it can be used to train clinicians and showcase new devices and procedural techniques.

### Limitations

While the simulator effectively replicates accurate pressure and inflow values and serves as a sophisticated tool for device development and procedural training, several limitations remain. First, the rigid LA model, while facilitating the rapid exchange of different LAA geometries, does not replicate the mechanical properties of native tissue, or account for individual differences in the structure and size of the LA. Future iterations could explore soft, compliant models or hybrid approaches that more closely resemble the native LA and are patient-specific. Second, the simulator lacks active atrial contraction, which prevents the replication of the atrial kick and the associated a- and A-waves in pressure and flow waveforms, respectively [49](Supplementary Fig. 9). Incorporating pneumatic actuators, like those used for LV [50] and RV [51] biomechanics, to simulate active contraction could provide valuable insights into device performance under dynamic atrial conditions. This enhancement could have significant implications for device placement, risk of device embolization, and long-term performance post-implantation. Third, the use of custom actuation to control atrial rhythms would allow for more accurate recreation of AF-specific dynamics rather than atrial flutter. Developing an entirely soft model capable of active contraction would enable the simulator to replicate not only target pressure and flow values but also precise waveforms and rhythms, achieving a higher-fidelity recreation of LA function. Fourth, although the LAA’s impact on hemodynamics is partly driven by its function as a capacitance chamber, its neurohormonal function also plays an important role [52]. However, given the complex nature of its neurohormonal function, this could not be replicated and examined in our current study. Fifth, this study evaluates only the WATCHMAN device and does not include other commonly used devices, such as the Amulet with its disc-and-lobe configuration, which may result in different hemodynamic changes following LAAO. Finally, although some PDLs are detected at the time of device deployment, a significant proportion develops later due to ongoing LA and LAA remodeling post-LAAO, presenting an unresolved issue with LAAO devices [18, 53, 54]. Nevertheless, our study focused exclusively on evaluating the use of the simulator in identifying PDLs during the procedure. Future studies assessing the utility of the patient-derived left atrial benchtop simulators in simulating LA and LAA remodeling and, thus, predicting post-procedural PDLs are needed.

## Conclusion

In summary, we present the development and validation of a modular, patient-derived left atrial benchtop simulator that can recreate hemodynamics of healthy adults and patients with atrial fibrillation. The simulator offers a highly realistic environment for the evaluation of LAAO devices, procedural planning, and operator training. By integrating patient-specific anatomical features with highly tunable hemodynamic parameters, the simulator provides valuable tools for testing new devices, refining transcatheter or surgical interventions and procedures, and improving clinician training. We believe this platform has the potential to improve patient outcomes by enhancing device design, optimizing procedural strategies, and reducing complications associated with LAAO interventions. Future efforts will focus on incorporating active atrial contraction and fully compliant left atrial models to further refine the simulator and expand its applications in cardiovascular research and clinical practice.

## Electronic Supplementary Material

Below is the link to the electronic supplementary material.

Supplementary Material 1

Supplementary Material 2
