# Supplementary material for: Design and Validation of a High-Fidelity Left Atrial Cardiac Simulator for the Study and Advancement of Left Atrial Appendage Occlusion
Source: Cardiovasc Eng Technol. 2025 Jan 27;16(3):279–95. doi: 10.1007/s13239-025-00773-2 (PMC12129853; doi:10.1007/s13239-025-00773-2)
Supplement: Supplementary file 1 — Supplementary Material 1 [file 13239_2025_773_MOESM1_ESM.docx]

**Title:** Design and validation of a high-fidelity left atrial cardiac simulator for the study and advancement of left atrial appendage occlusion.

Updated and/or new figures/tables are highlighted in yellow.

Changes to figure/table captions are written in blue font.

**Supplementary Information:**

**Supplementary Table 1.** Endovascular left atrial appendage closure devices.

**
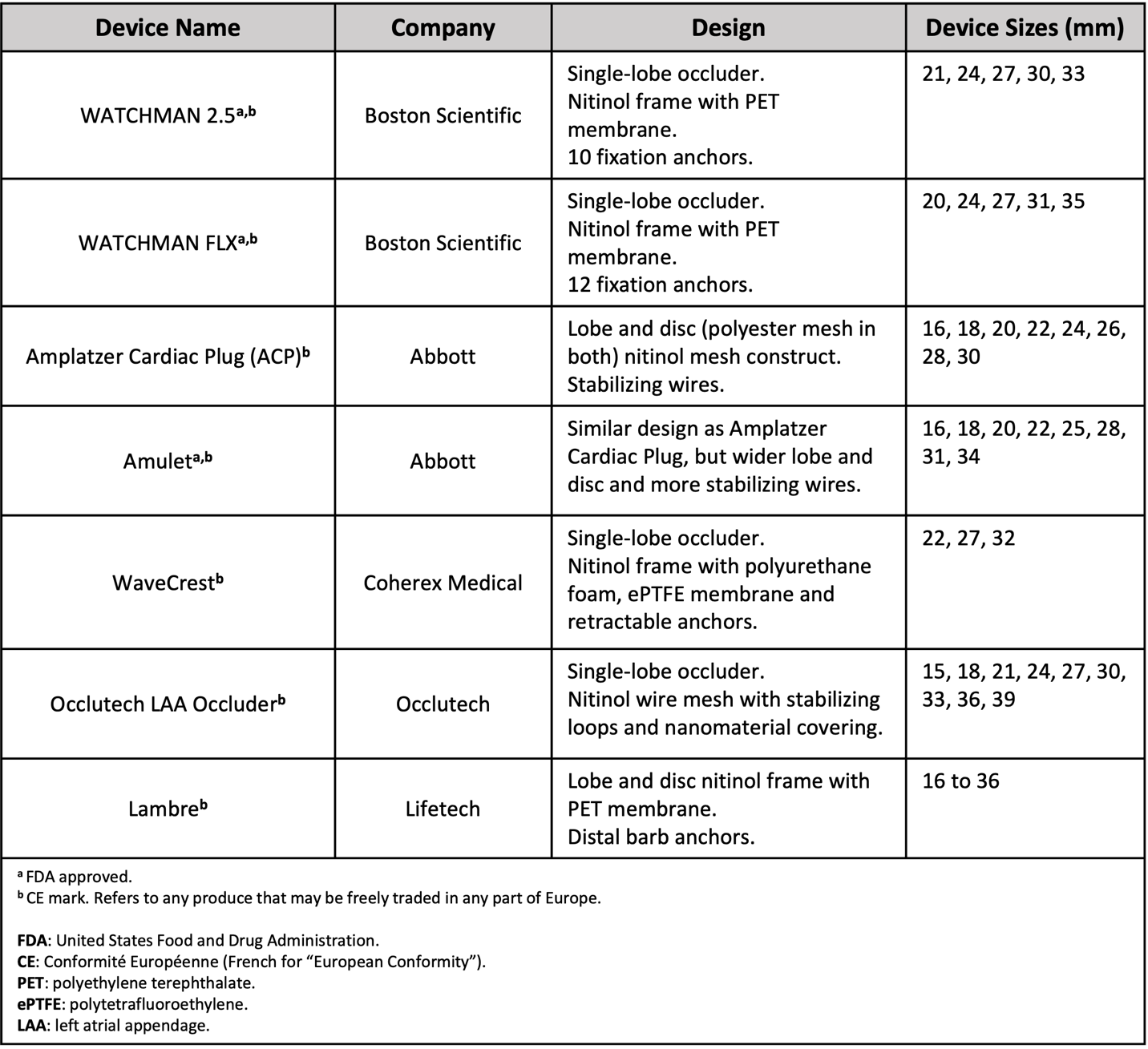
**

**Supplementary Table 2.** Benchmark pressure data for validation of LA cardiac simulator.


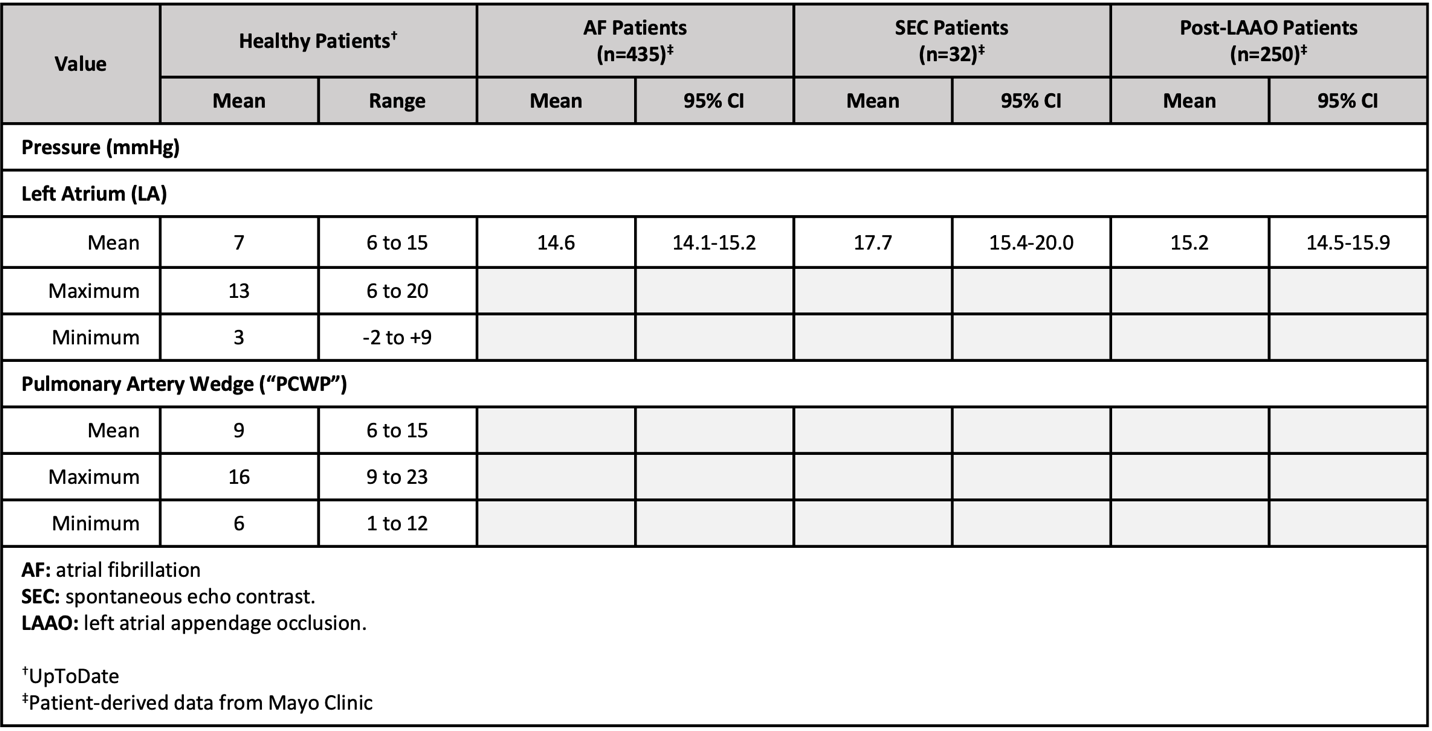


**Supplementary Table 3.** Benchmark flow data for validation of LA cardiac simulator.

**
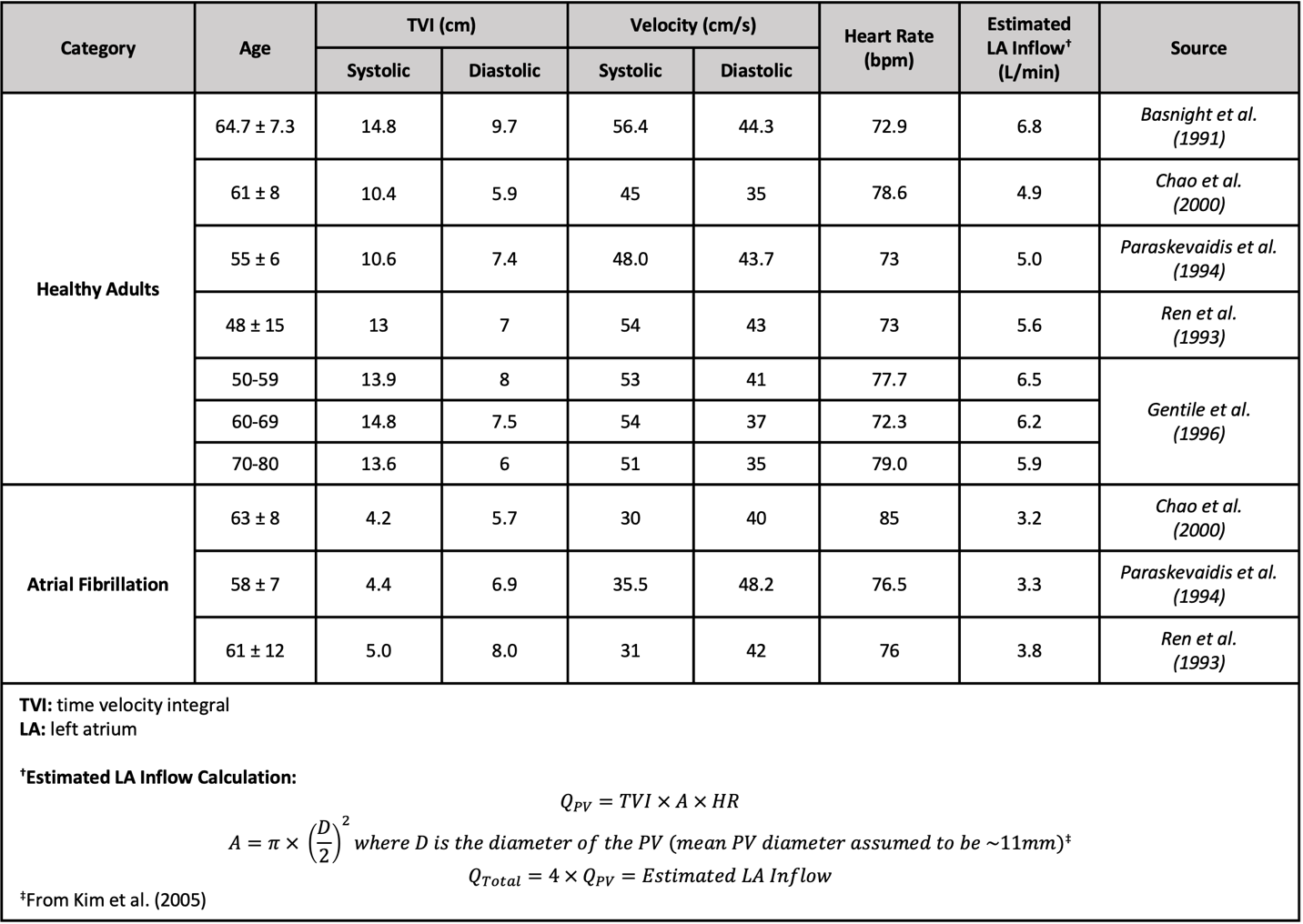
**

**Supplementary Table 4.** Hemodynamic values of normal recumbent adults. Values from UpToDate: <https://www.uptodate.com/contents/pulmonary-artery-catheterization-interpretation-of-hemodynamic-values-and-waveforms-in-adults>

**
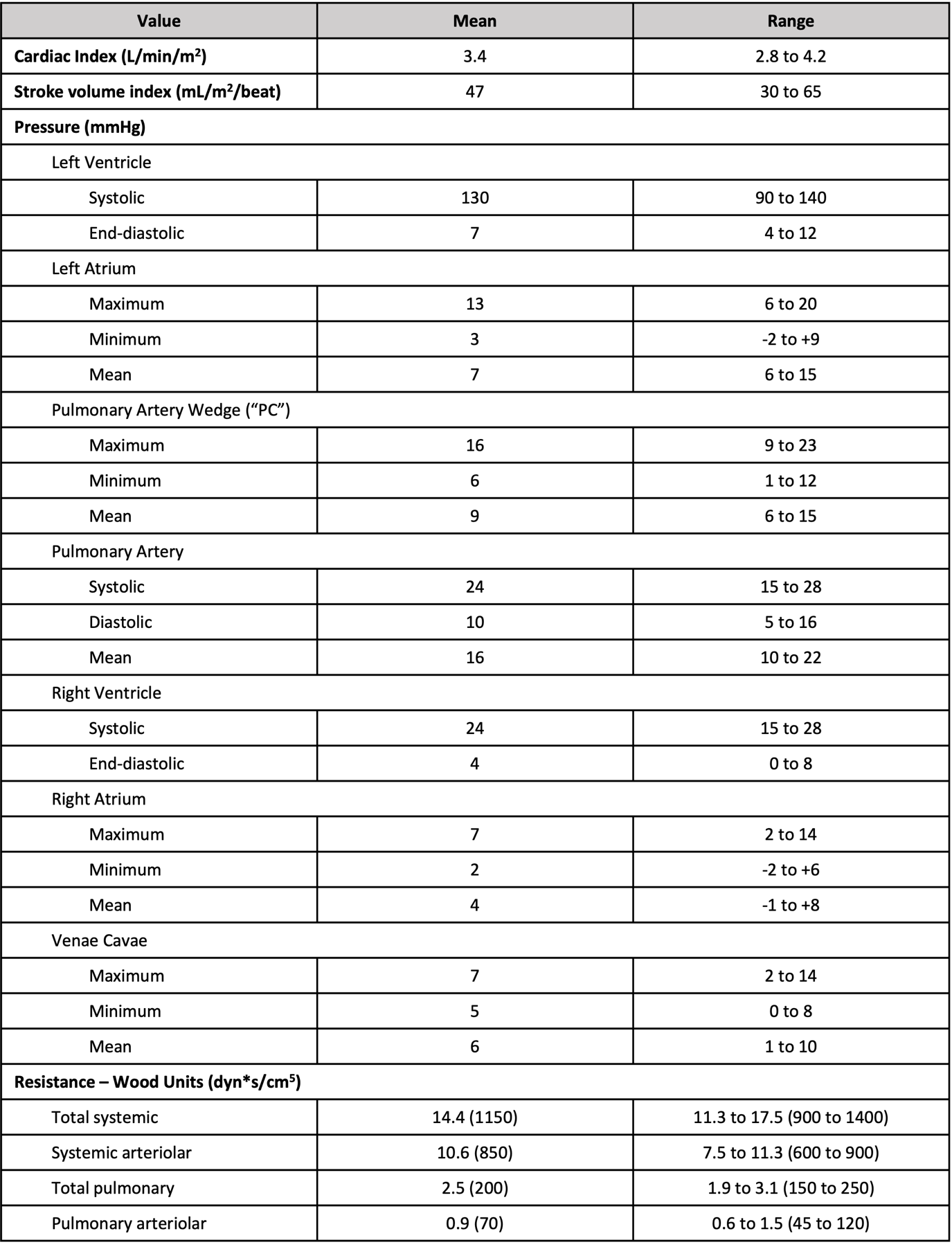
**

**Supplementary Table 5.** Pressure values in healthy patients and patients pre- and post-LAAO. Values from Mayo Clinic.

**
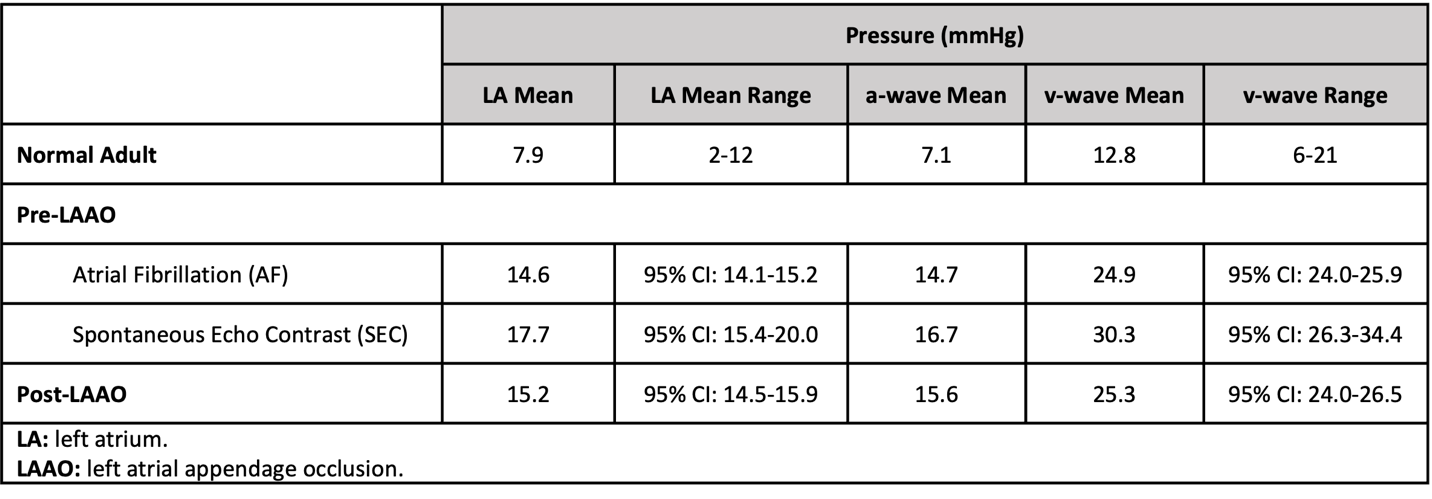
**

**Supplementary Table 6.** Velocity values in healthy patients and patients pre-LAAO. Values from Mayo Clinic.

**
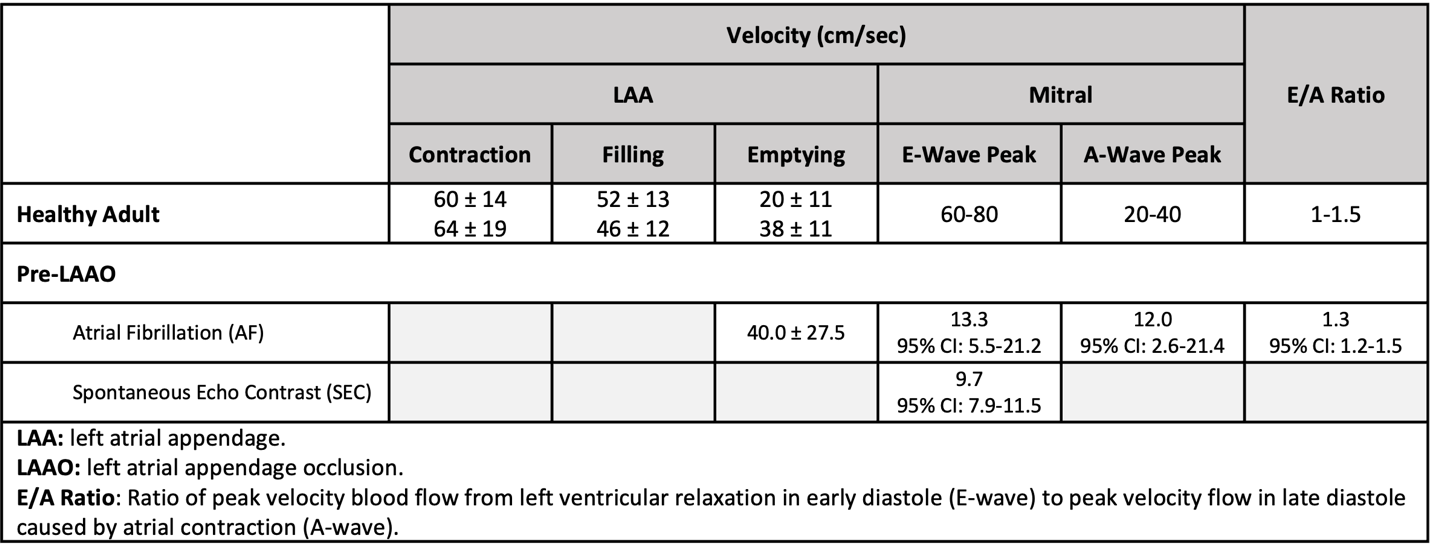
**

**
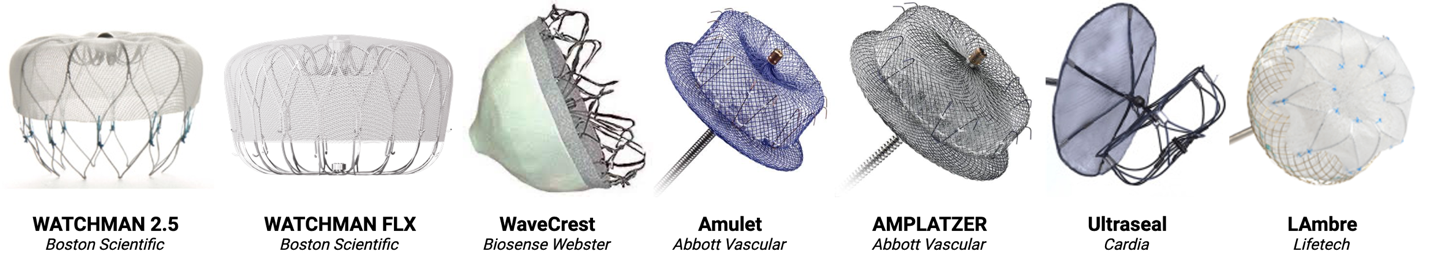
**

**Supplementary Figure 1.** Commercially available, CE-marked percutaneous LAAO devices.

**
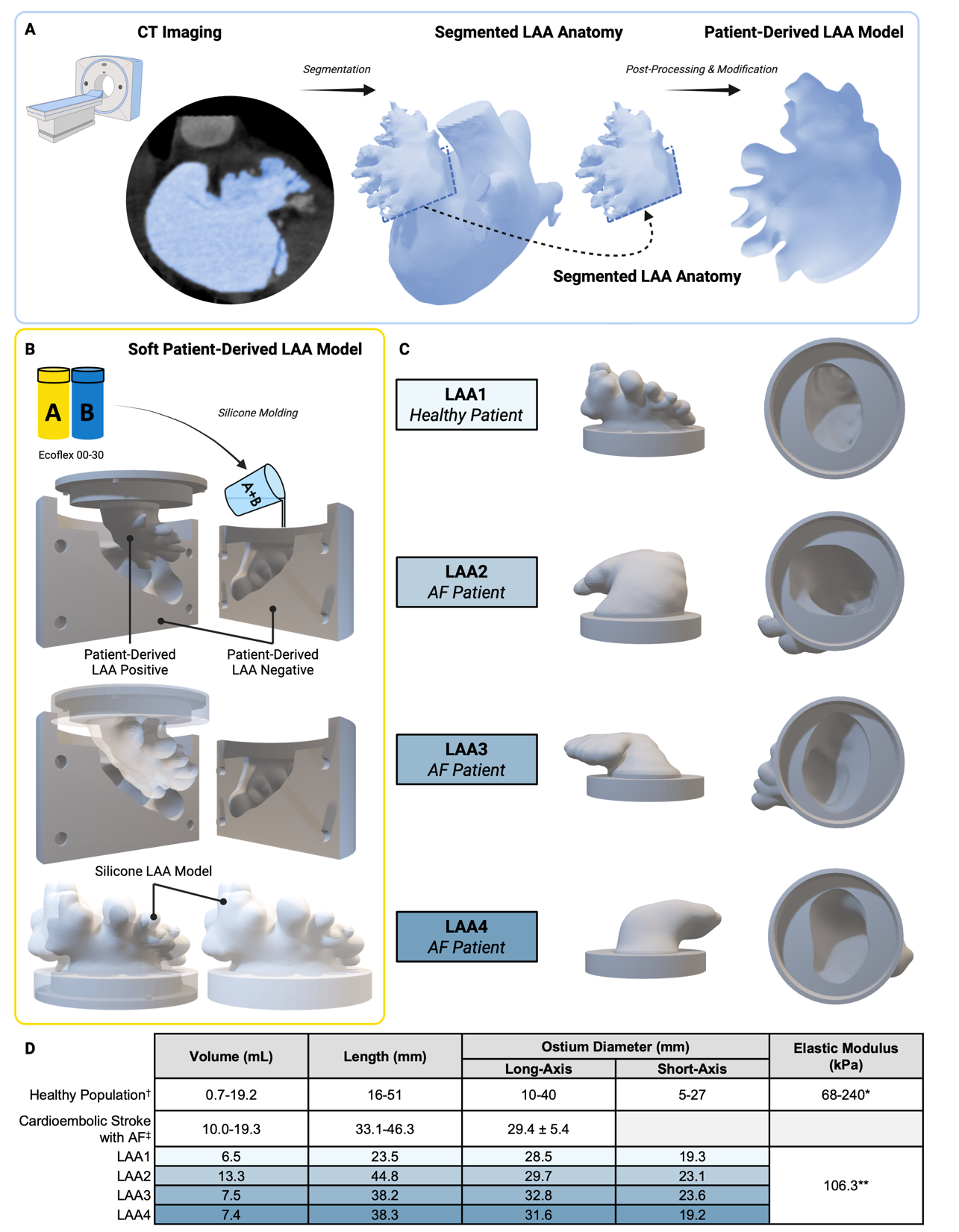
**

**Supplementary Figure 2. Workflow from clinical imaging to soft, patient-derived LAAs for attachment to rigid model. (A)** Image segmentation, post-processing, and cutting at the ostium plane. **(B)** 3D printed mold for silicone casting of LAA geometry. **C.** Library of soft, patient-derived LAA models. *Healthy* indicates LAA from healthy patient. *AF* indicated LAA from patient with atrial fibrillation. AF: atrial fibrillation. **(C)** Table comparing measurements of the LAA models with the healthy population and patients with AF who experienced cardioembolic stoke. ^†^Values for Healthy Population from Al-Saady et al.^1^ ^‡^Values for Cardioembolic Stroke with AF (n=57) from Jeong et al.^31^ *Elastic modulus of real LAA tissue at ε=10-20% from Fanni et al.^27^ **Elastic modulus of 2mm thickness Ecoflex 00-30.


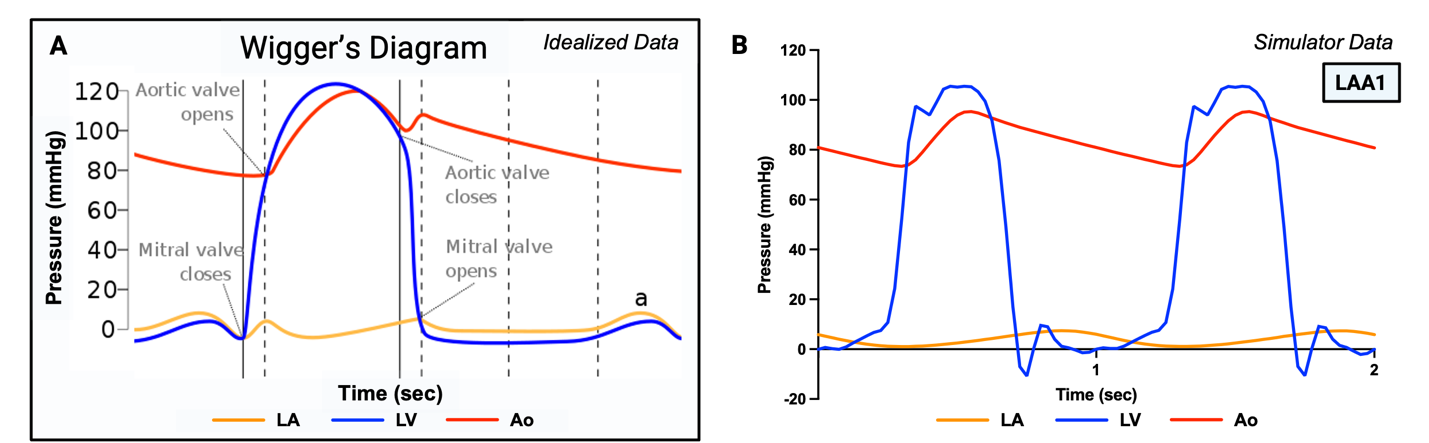


**Supplementary Figure 3. Replication of Wiggers Diagram with LA cardiac simulator. (A)** Wiggers Diagram showing idealized pressure curves in the left atrium (LA), left ventricle (LV) and aorta (A_o_) over a single cardiac cycle. **(B)** Physiological pressure curves in the left atrium (LA), left ventricle (LV) and aorta (A_o_) over two cardiac cycles measured in the circulatory flow loop. Data collected at 60 BPM and 75 mL stroke volume using LAA1.


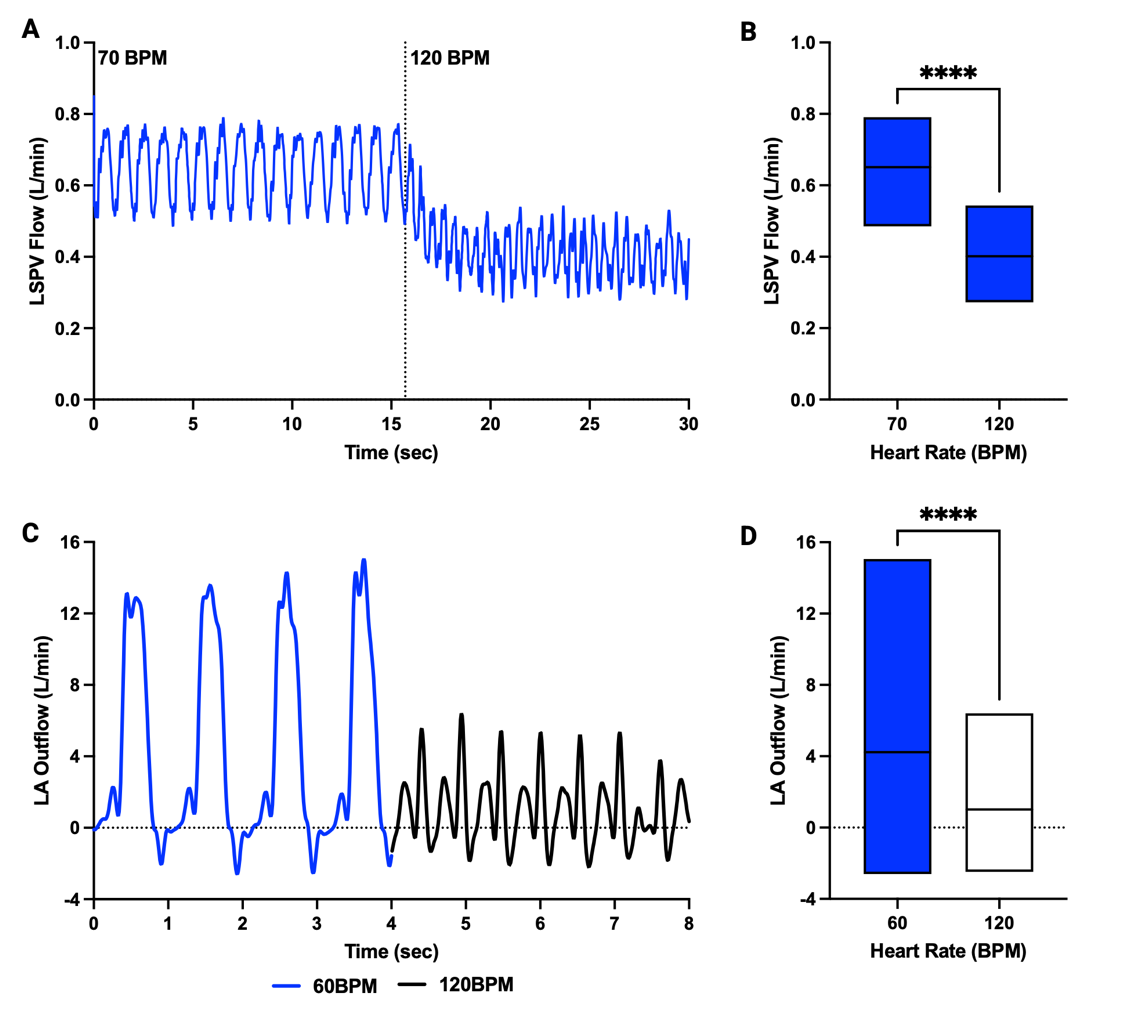


**Supplementary Figure 4. Simulation of AF hemodynamics by increasing pump heart rate. (A)** LSPV flow measured in cardiac simulator as pump HR is increased from 70 BPM to 120 BPM in real time. **(B)** Mean LSPV flow measured in cardiac simulator over 10 seconds as pump HR is increased from 70 BPM to 120 BPM to mimic AF. **(C)** LA outflow measured in cardiac simulator at pump HR of 60 BPM and 120 BPM. **(D)** Mean LA outflow measured in cardiac simulator over 10 seconds at pump HR of 60 BPM and 120 BPM to mimic AF. Welch’s two-tailed t-test, **** <0.0001. LSPV: left superior pulmonary vein; LA: left atrium; HR: heart rate.


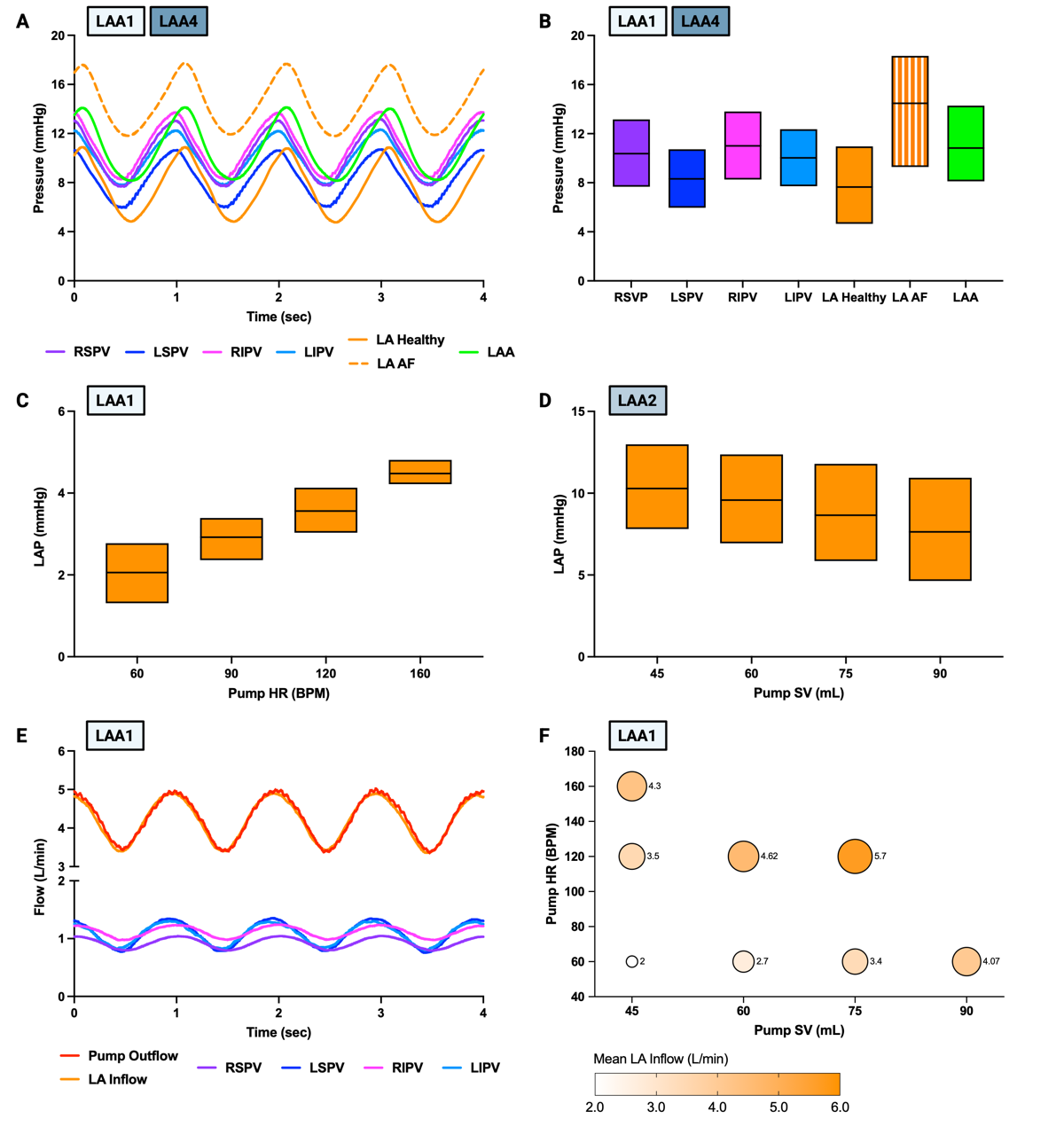


**Supplementary Figure 5. Tunability of pressures and flows measured in LA cardiac simulator.** **(A)** (Patho)physiological pressures measured in LA cardiac simulator. Pressure in the four pulmonary veins, the left atrium (LA) and the left atrial appendage (LAA). The simulator can be tuned to achieve physiological LA pressures (LA Healthy), and pathological pressures observed in patients with atrial fibrillation (LA AF). LA AF data collected with LAA4. All other data collected with LAA1. All data collected at 60 BPM. **(B)** Mean pressures measured in LA cardiac simulator over 10 cardiac cycles. Maximum and minimum pressures indicated by upper and lower bounds of box, respectively. LA AF data collected with LAA4. All other data collected with LAA1. All data collected at 60 BPM. **(C)** Mean LAP measured in LA cardiac simulator over 10 cardiac cycles as pump HR is increased from 60 BPM to 160 BPM. Maximum and minimum pressures indicated by upper and lower bounds of box, respectively. All data collected with LAA1 at 45 mL pump SV. **(D)** Mean LAP measured in LA cardiac simulator over 10 cardiac cycles as pump SV is increased from 45 mL to 90 mL. Maximum and minimum pressures indicated by upper and lower bounds of box, respectively. All data collected with LAA2 at 60 BPM. **(E)** Flows measured in LA cardiac simulator. Total pump outflow, flow through the four pulmonary veins and LA inflow measured as sum of flow through the pulmonary veins. All data collected with LAA1 at 60 BPM. **(F)** Tunable LA inflow by varying pump HR and pump SV. RSPV: right superior pulmonary vein; RIPV: right inferior pulmonary vein; LIPV: left inferior pulmonary vein; LSPV: left superior pulmonary vein; LA: left atrium; LAA: left atrial appendage; HR: heart rate; SV: stroke volume.


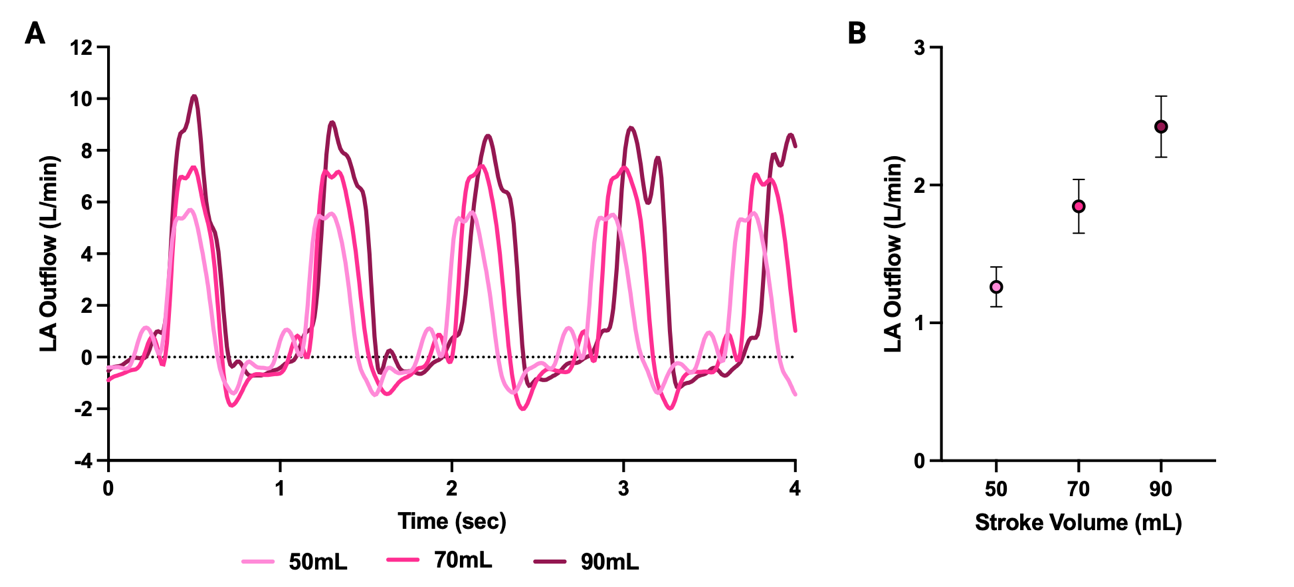


**Supplementary Figure 6. Tunability of LA outflow. (A)** Tunable LA outflow by varying pump SV. **(B)** Mean LA outflow measured in cardiac simulator over 10 cardiac cycles as pump SV is increased from 50 mL to 90 mL. Maximum and minimum flows indicated by upper and lower bounds of box, respectively. All data collected with LAA1 at 70 BPM. LA: left atrium.


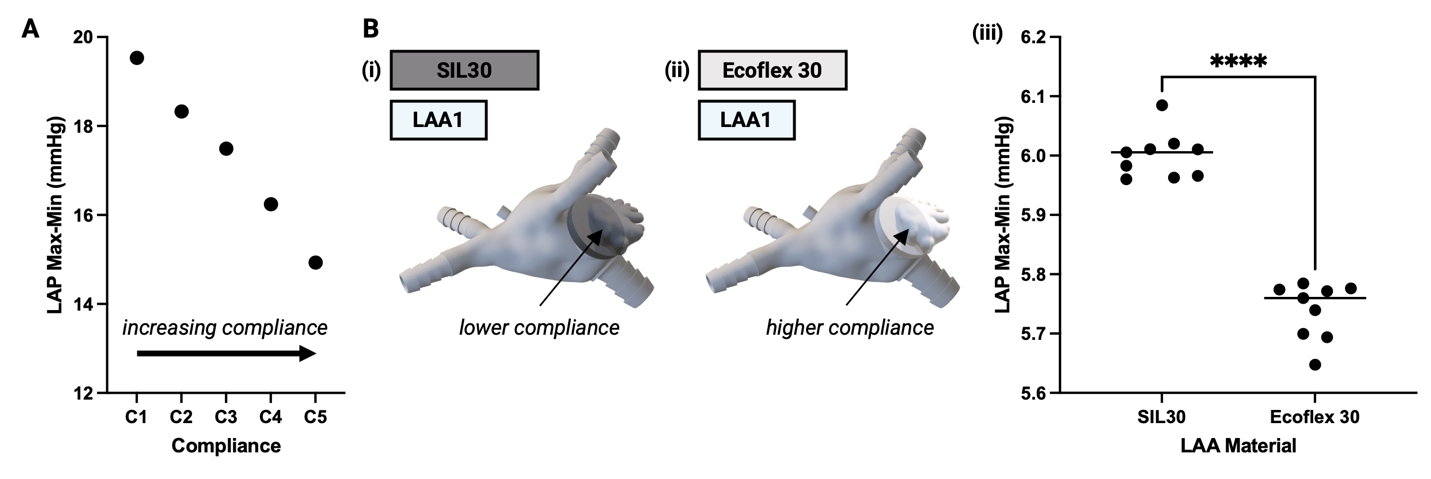


**Supplementary Figure 7. Hemodynamics can be tuned by varying compliance through either the Windkessel elements or the material properties of the LAA model. (A)** LA pressure amplitude (LAP_PulsePressure_ = LAP_maxima_ – LAP_minima_) as compliance is increased from C1 (lowest compliance) to C5 (highest compliance). Compliance can be tuned by varying the height of the liquid-air interface in the compliance chambers. Data collected with LAA1 (SIL30) at 60 BPM and pump SV of 90 mL. **(B)** Varying compliance of LAA model. (i) Lower compliance LAA model 1 (SIL30). (ii) Higher compliance LAA model 1 (Ecoflex 30). (iii) LA pressure amplitude (LAP_PulsePressure_ = LAP_maxima_ – LAP_minima_) for different models. Data collected at 60 BPM and pump SV of 90 mL. Welch’s two-tailed t-test on n=9 heartbeats from each model under the same simulator conditions (pump HR and SV, circuit resistance, circuit compliance), **** <0.0001. LAP: left atrial pressure; LAA: left atrial appendage; HR: heart rate; SV: stroke volume.

**
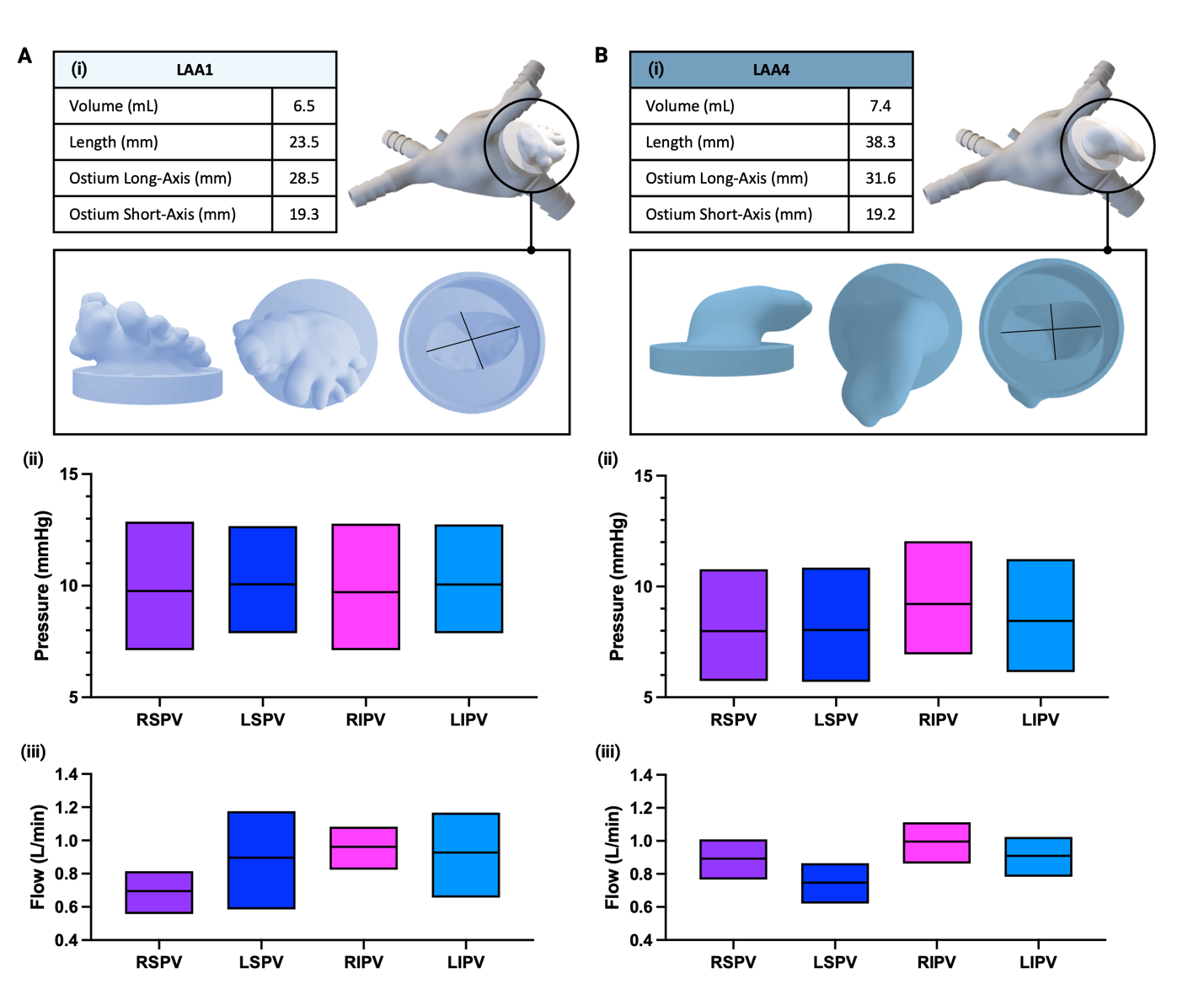
Supplementary Figure 8. Variable hemodynamics with different LAA model geometries. (A)** LAA Model 1. (i) Model characteristics. (ii) Mean pulmonary venous pressure over 5 cardiac cycles. Maximum and minimum pressures indicated by upper and lower bounds of box, respectively. (iii) Mean pulmonary venous flow over 5 cardiac cycles. Maximum and minimum flows indicated by upper and lower bounds of box, respectively. Data collected at 60 BPM and pump SV of 75 mL. **(B)** LAA Model 4. (i) Model characteristics. (ii) Mean pulmonary venous pressure over 5 cardiac cycles. Maximum and minimum pressures indicated by upper and lower bounds of box, respectively. (iii) Mean pulmonary venous flow over 5 cardiac cycles. Maximum and minimum flows indicated by upper and lower bounds of box, respectively. Data collected at 60 BPM and pump SV of 75 mL. RSPV: right superior pulmonary vein; RIPV: right inferior pulmonary vein; LIPV: left inferior pulmonary vein; LSPV: left superior pulmonary vein; LAA: left atrial appendage; SV: stroke volume.

**
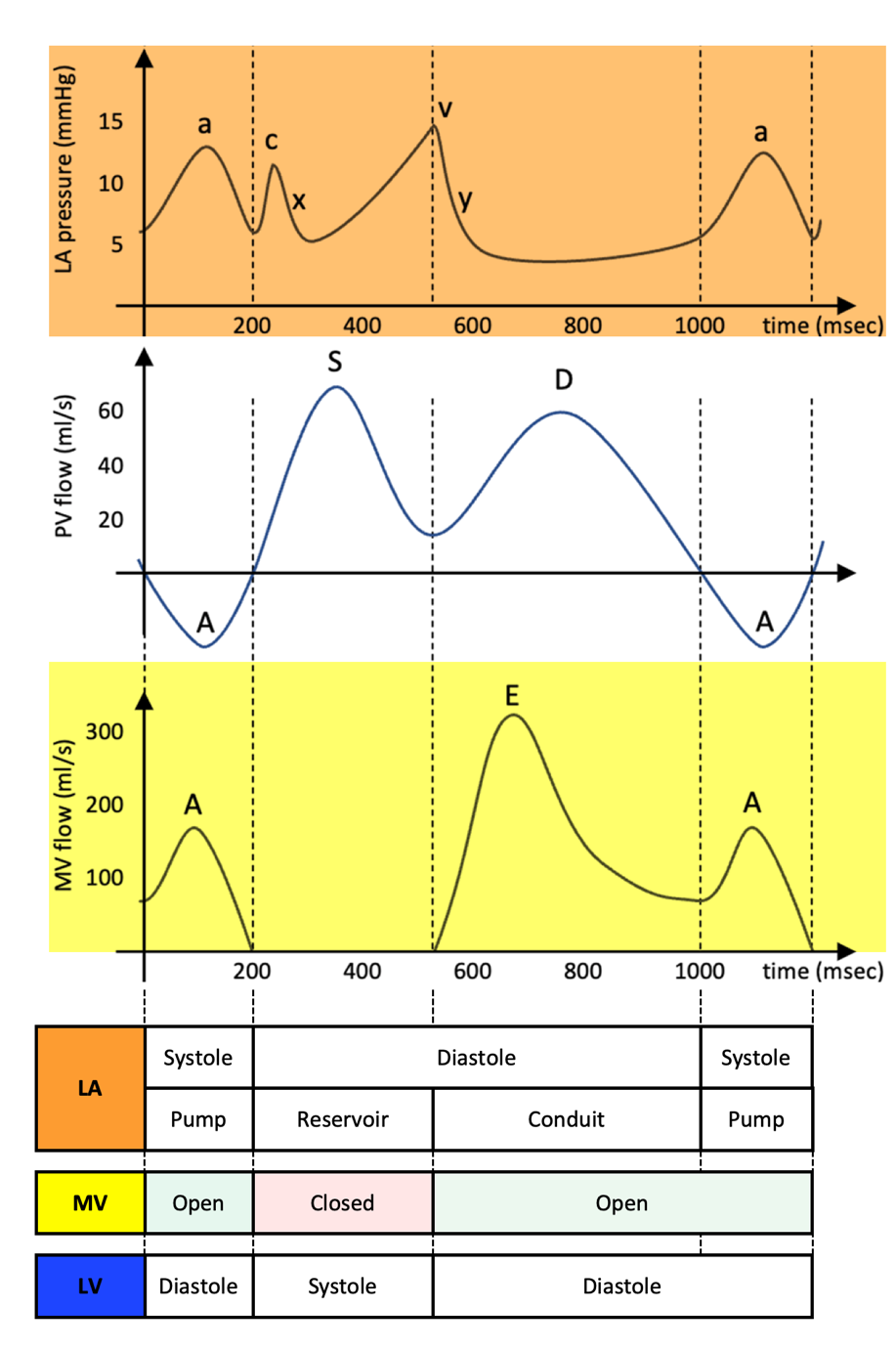
**

**Supplementary Figure 9.** Relationship between left atrial pressure, pulmonary venous flow, and mitral valve flow with respect to the left atrial and left ventricular phases of the cardiac cycle, along with the state of the mitral valve. Adapted from Meskin et al.^54^
