# Supplementary material for: Design and Validation of a High-Fidelity Left Atrial Cardiac Simulator for the Study and Advancement of Left Atrial Appendage Occlusion
Source: Cardiovasc Eng Technol. 2025 Jan 27;16(3):279–95. doi: 10.1007/s13239-025-00773-2 (PMC12129853; doi:10.1007/s13239-025-00773-2)
Supplement: Supplementary file 2 — Supplementary Material 2 [file 13239_2025_773_MOESM2_ESM.docx]

**Title:** Design and validation of a high-fidelity left atrial cardiac simulator for the study and advancement of left atrial appendage occlusion.

Author responses are written in purple font.

**COMMENTS FOR THE AUTHOR:

Editors:** 

Dear authors, 

Your manuscript was reviewed by three experts who found your work interesting but have identified a number of limitations that need to be addressed:

1. The validation of the simulator is not sufficiently detailed, making it difficult to assess its accuracy and reliability in replicating physiological conditions. I think it would be a good idea for the authors to provide a more comprehensive explanation of their validation methods and the parameters used in the experiments.
2. There are multiple instances where the methodology lacks clarity, particularly in describing the selection criteria for anatomical models and the setup of the mock circulatory loop. Ensuring that these details are explicitly addressed is crucial for reproducibility.
3. There are inconsistencies in the results as presented, particularly concerning the pressure and flow waveforms. It is essential for the authors to clarify these discrepancies and ensure that the data aligns with physiological expectations.

We sincerely thank you for the opportunity to revise our manuscript and for summarizing the key areas for improvement. We have carefully addressed the feedback provided by the reviewers and your comments, and we believe the revised manuscript is significantly strengthened as a result. Below, we outline how we have addressed the specific concerns you raised:

1. **Inclusion of Additional Authors**: We have added two clinicians, Mohamad Alkhouli, MD, and Hasan Alarouri, MD, as co-authors. Both are experienced practitioners of the LAAO procedure and have reviewed and supported this work. Their expertise underscores the clinical relevance of the simulator and its alignment with real-world practice.
2. **Enhanced Validation of the Simulator**: To address concerns regarding the validation of our simulator, we have conducted additional validation experiments and provided a more comprehensive explanation of our validation methods and parameters. We have included a new figure (Figure 3) that presents clear and concise validation results, demonstrating that the simulator replicates both healthy and pathological conditions accurately. Supplementary materials now include detailed comparisons of pressure and flow values against established references and clinical data, ensuring the reliability and accuracy of our system are thoroughly conveyed.
3. **Improved Methodological Clarity**: We recognize the critical importance of methodological clarity for reproducibility and have revised the methods section accordingly. Specific updates include a detailed explanation of the selection criteria for anatomical models, additional details and rationale describing modifications to the left atrium model, such as the attachment feature and the septal crossing window, and a clearer, more comprehensive description of the mock circulatory loop setup. This now includes explicit reasoning behind specific design choices, such as the placement of resistance and compliance elements to mitigate non-physiological suction effects. Additionally, we have included photographs of the complete experimental setup to provide visual context, further enhancing understanding.
4. **Resolution of Inconsistencies in Results**: We have addressed inconsistencies in the pressure and flow waveforms and clarified these discrepancies throughout the manuscript. Specifically, we have emphasized mean values rather than waveform morphology to align better with physiological expectations. We also ensured that flow and pressure data aligned with established references.

We believe these revisions, along with the addition of clinical authors, comprehensively address the limitations identified by you and the reviewers. The manuscript now demonstrates greater methodological clarity, rigorous validation of the simulator, and alignment of results with physiological expectations, ensuring that it meets the high standards of your journal. We thank you for your constructive feedback and look forward to your further evaluation. Please do not hesitate to contact us if further clarifications or revisions are required.

**Reviewer #2:** This manuscript presents a left atrial simulator designed for the study of left atrial appendage occlusion. The simulator integrates three-dimensional printing with a mock circulatory loop to reproduce the left atrial appendage occlusion procedure and evaluate the fluid dynamic modifications associated with the occlusion. The authors detail the design of the left atrial model, allowing for the adoption of various left atrial appendage geometries, and provide a description of the mock circulatory loop employed, along with experiments conducted to validate the system and demonstrate its tunability. Although the proposed simulator is innovative, the results pertaining to the validation and tuning processes are incomplete and occasionally conflicting. The manuscript requires several corrections and improvements before it can be considered for publication. Please see my detailed comments below.

We thank Reviewer #2 for their thoughtful and detailed comments and suggestions, which have significantly strengthened the manuscript. Their insights have guided meaningful revisions, including the addition of new data to validate the simulator, enhanced clarity in the methods section, and improved rationale for experimental design. We have addressed the concerns regarding conflicting data by clarifying that these results were collected under different experimental conditions and simulator settings in our attempt to demonstrate the versatility and tunability of the simulator. To ensure clarity, we have provided more detailed explanations and modified figures where necessary to present a more consistent and coherent message, reducing the potential for confusion among readers. We believe these revisions have meaningfully addressed Reviewer #2’s concerns and have significantly improved the overall quality, clarity, and rigor of the manuscript. Detailed, point-by-point responses to each of the Reviewer’s comments are provided below.

**Introduction:**

a) The introduction lacks citation of existing 3D-printed simulators for LAAO. I recommend including the recent work by Fanni et al. ("A high-fidelity personalised 3d printed simulator for the left atrial appendage occlusion procedure") as it presents a relevant foundation.

We thank the Reviewer for highlighting this important work. We agree it provides a relevant foundation for the field. We have added this reference to the introduction and emphasized how our work builds upon these advances while addressing the need for incorporating dynamic fluid flow and tunable hemodynamic conditions.

b) The authors introduce finite element analysis for the study of device deployment; however, they do not cite any relevant literature. I suggest including the following reference, which specifically studied the Watchman FLX device: Danielli, Francesca, et al. "Left atrial appendage occlusion: On the need of a numerical model to simulate the implant procedure." International Journal for Numerical Methods in Biomedical Engineering (2024): e3814.

We appreciate this suggestion and have included the recommended citation to underscore the utility of numerical modeling in simulating device deployment. This addition strengthens the discussion of computational approaches as a complementary tool in LAAO device evaluation.

**Methods**

c) Selection criteria for the left atrium (LA) from the database of 100 patients are unclear. Please clarify if this was an averaged geometry or based on specific anatomical criteria.

We apologize for the lack of clarity in the initial submission. We have revised the methods section to specify that a single patient geometry was selected based on anatomical criteria, including left atrial dilation and a long-axis dimension exceeding 32 mm, representative of patients with atrial fibrillation and stroke risk.

d) Key dimensions, including the sizes of the LAA connector, barbed connectors, septal crossing, and LA model thickness, are missing. Providing these details would enhance replicability.

We thank the Reviewer for highlighting this omission. We have now included all relevant dimensions for the LAA connector, barbed connectors, septal crossing, and LA model thickness in the revised methods section to enhance replicability.

e) The thickness of the LA model was not reported in the text.

We apologize for this oversight and have now included the thickness of the LA model in the revised methods.

f) I suggest describing the design of the septal crossing, including any geometric or material considerations.

We appreciate the Reviewer’s recognition of the importance of this design element. In response, we have expanded the description in the Methods section to address both geometric and material considerations in the design of the septal crossing. Specifically, the septal crossing window was designed with clinician input to capture an anatomically appropriate location for this particular LA geometry while remaining versatile for use with other LAA geometries. The design incorporates modularity to allow varied crossing angles, accommodating multiple LAA configurations. This ensures adaptability across different procedural requirements. Although optimizing a realistic transeptal puncture (TSP) was not the primary objective of this study, we acknowledge its critical role in clinical procedures and have identified this as a promising area for future improvement.

g) Include information about the pressure connector (size, type) used in the LA model.

The type, size, and location of the pressure connectors have been detailed in the revised methods to improve clarity and replicability.

h) Clearly state the anatomical variations represented (e.g., cauliflower, windsock, chicken wing, cactus) and the selection criteria for these LAA models.

We appreciate the reviewer’s emphasis on the importance of providing clear selection criteria, and we apologize for the initial lack of detail in this regard. While these naming conventions for LAA morphologies (e.g., cauliflower, windsock, chicken wing, cactus) are widely recognized, we believe that they are often subjective and overlapping. Nicol et al. recently highlighted in JCCT^1^ that these definitions lack precise classification, as the commonly used subtypes often exhibit significant variability and unclear boundaries. To avoid potential ambiguity, we have removed these traditional designations from the manuscript. Instead, we have included detailed dimensions and morphological characteristics of the selected LAA models, which are now described in the Methods section and summarized in Supplementary Figure 2. We believe this approach provides a more objective and precise characterization of the anatomical variations represented in our study.

i) Describe how the LAA geometry was adjusted to connect with the LA model, as this is a critical aspect of simulator assembly.

We thank the Reviewer for highlighting this critical aspect of simulator design. We have provided additional details on how the LAA geometry was adjusted to ensure proper attachment to the LA model, particularly focusing on the preservation of the ostial landing zone for device deployment.

j) Provide a rationale for using Ecoflex for all geometries and SIL30 for one specific model, supported by relevant literature on how these material properties impact occlusion performance.

We appreciate the opportunity to clarify this design choice. Ecoflex was selected as the primary material due to its translucency, which facilitates visualization during experiments, and its ease of manufacturing, which enables consistent fabrication of patient-specific models. SIL30 (Carbon Inc) was utilized for one specific geometry to demonstrate an alternative manufacturing approach with superior resolution and increased stiffness, reflecting potential pathological compliance changes such as those observed in atrial fibrillation or fibrosis. Tensile testing informed the thickness of the Ecoflex models to ensure they aligned with the elastic properties of native tissue as reported in the literature. The rationale for these material choices, along with supporting references, has been added to the Methods section for clarity.

k) The rationale behind the selected components in the mock circulatory loop (MCL) setup requires further explanation. For example, the authors incorporated two compliance chambers and a resistance between the mitral valve and the LA model. This configuration likely impacts the mitral flow waveform imposed on the LA model and influences pressure within the LA. Explaining the function of each component and how it contributes to mimicking physiological flow conditions would improve clarity. Additionally, the configuration of the pulmonary vein connection to the reservoir, which uses a 4-to-1 adapter, a single tube, resistance, and several adapters, may introduce significant pressure losses. Given this setup, it is unclear if the system can effectively replicate both physiological mitral flow and atrial pressure (approximately 4 mmHg) simultaneously. The authors must clarify how these elements were selected to address the LA boundary conditions.

We thank the Reviewer for highlighting the need for additional explanation. We have expanded the Methods section to provide a detailed rationale for each component in the MCL setup, including the compliance chambers, resistance valves, and the 1:4 adapter. Specifically, we explain how the compliance chambers and resistance valves allow for fine-tuning of mean LAP and LA inflow/outflow, creating physiologically relevant pressures and flow conditions. Preliminary testing confirmed that the system maintained mean LA pressures in the healthy physiological range of 5-15 mmHg. To achieve these results, we carefully adjusted the compliance chamber volumes and resistance values through iterative design, balancing system complexity with physiological accuracy. We also address the role of the 1:4 adapter, emphasizing its larger diameter to minimize pressure losses and its function in ensuring even distribution of pulmonary vein inflow. While the focus of this study was primarily on replicating mean LAP and inflow/outflow rather than specific waveform fidelity, we acknowledge the limitations of our system in reproducing detailed mitral flow waveforms. This limitation arises partly due to the lack of active control over atrial contraction. We have noted this as a limitation in the Discussion section and identified it as an area for future improvement, particularly with potential incorporation of active atrial contraction.

l) The tubes diameter and thickness must be provided.

Tubing dimensions and part numbers have been specified in the revised methods.

m) Specify the type of pressure sensor used for measuring left atrial pressure.

The type of pressure sensor has been specified in the revised methods section.

n) A picture of the overall MCL is missing.

A photo of the complete MCL setup has been included in the updated Figure 2 for clarity.

o) The measurement of cardiac output (CO) in the mock circulatory loop appears to be taken at the outlet of the pulmonary veins, which could lead to inaccuracies due to the influence of compliance chambers and tubing along the flow path. To provide a more accurate assessment of CO, I recommend measuring flow directly at the left atrium (LA) model outlet, where flow conditions are less affected by downstream components. This adjustment would offer a more reliable representation of the physiological cardiac output and ensure that flow patterns imposed on the LA model align with expected values.

We greatly appreciate this insightful comment and the opportunity to strengthen the study through additional data collection. Following the Reviewer’s recommendation, we have measured flow directly at the LA model outlet to provide a more accurate representation of flow conditions. These new measurements have been incorporated into the updated manuscript (Figure 4 and Supplementary Figure 6). Additionally, we have clarified the terminology used in the manuscript to avoid potential confusion. Specifically, the term “cardiac output (CO)” has been replaced with more precise descriptions: “LA inflow” (sum of flow from the pulmonary veins), “LA outflow” (flow measured directly at the LA model outlet), and “total flow” (flow measured directly at the pulsatile pump outlet). We believe this adjustment improves the clarity and rigor of the results, addressing the reviewer’s concern and ensuring the data align with physiological expectations.

p) The authors should specify which pressure and flow measurements were used to assess hemodynamic changes due to LAA occlusion.

We apologize for the lack of specificity in the initial descriptions. The revised manuscript now clarifies the pressure and flow metrics used to assess hemodynamic changes due to LAAO. Specifically, we distinguish between “LA inflow” (sum of flow from the pulmonary veins), “LA outflow” (flow measured directly at the LA model outlet), and “total flow” (flow measured directly at the pulsatile pump outlet). Additionally, we have noted that certain measurements are research-specific metrics derived from the simulator rather than clinically utilized parameters. These metrics serve as measurable indicators of occlusion effectiveness and device performance within the context of this study. We hope these updates enhance the clarity and comprehensiveness of the manuscript.

q) The authors should clarify the purpose of the occlusion device coating. In particular if this is intended to simulate platelet coating.

We thank the reviewer for this suggestion. We have clarified the purpose in the methods. The coating is not intended to simulate a clinical application but to create an impermeable device for research purposes, mimicking post-endothelialization behavior.

r) The M&M section is vague about device positioning and implantation techniques. Explain if deliberate mispositioning was employed and whether the Watchman delivery system was used via the septal crossing.

We agree with the reviewer about this lack of clarity. We have clarified that deliberate mispositioning was employed to simulate peri-device leak and that the Watchman delivery system was used during procedural training experiments.

s) Given the not planar geometry of the model, the authors should describe how the ultrasound (US) probe was positioned and any interface media used to facilitate ultrasound propagation.

We have clarified in the revised manuscript that the model was submerged in a water bath to optimize ultrasound imaging by facilitating consistent ultrasound propagation. The ultrasound probe was placed directly in contact with the submerged model and manipulated by a clinician to obtain optimal visualization and imaging angles.

**Results**

t) I suggest demonstrating intra-patient heterogeneity explicitly in the LAA models.

We appreciate the Reviewer’s comment and agree that intra-patient heterogeneity is a critical feature of our models. To address this, we have added detailed dimensions for the LAA models in the Methods section, summarized the range of heterogeneity in Supplementary Figure 2, and shown direct comparisons of geometries in Supplementary Figure 6. Additionally, we utilized different LAA geometries in various figures throughout the manuscript (e.g., LAA1 and LAA4 in Figure 3, LAA2 in Figure 4). These updates highlight the diversity and modularity of the system.

u) The validation process described in the manuscript lacks detail, making it difficult to assess the accuracy of the model in replicating physiological and pathological conditions. I recommend providing a more comprehensive description of the validation procedure, including the target mitral flow and pressure waveforms, specific resistance and compliance settings, parameters set on the pulsatile pump (e.g., cardiac output, beats per minute), and the height of the free fluid level in the reservoir. Additionally, it would be helpful to summarize these parameters in a table for quick reference.

We appreciate the reviewer’s comment and acknowledge the importance of providing a comprehensive description of the validation procedure. In response, we have significantly revised and expanded the manuscript to address these concerns. We have included a new Figure 3, which validates left atrial pressure (LAP) and left atrial inflow (LA inflow) against benchmark data from healthy patient reference values and clinical data from patients with atrial fibrillation. Supplementary Tables 2 and 3 summarize these benchmark pressure and flow data. Additional clinical reference data are provided in Supplementary Tables 4-6 for further context. The simulator was tuned to achieve target hemodynamic values using the pulsatile pump parameters (heart rate and stroke volume), compliance chamber settings, and static reservoir height. Where relevant, we have provided these parameters for clarity. However, we do not report fixed resistance, compliance settings, or fluid height values because the simulator is highly modular and designed to be adjusted to match desired physiological targets prior to each experiment. The simulator is a versatile tool that can be modified to suit various experimental needs. The modularity of the system allows users to fine-tune it to match their experimental goals. The compliance settings and open reservoir heights can be easily marked and logged for each experiment for consistent recalibration.

v) Indicate the parameters used to simulate atrial fibrillation conditions.

We thank the Reviewer for highlighting this important point. We have added more detailed explanations regarding how atrial fibrillation conditions were simulated. Specifically, we utilized patient-derived LAA geometries (LAA2, LAA3, LAA4) that reflect pathological features commonly associated with AF, such as increased volume. To replicate AF hemodynamics, we increased left atrial pressure (LAP) and heart rate. However, we acknowledge the limitation of our pulsatile pump, which generates a regular rhythm that more closely resembles atrial flutter than the “irregularly irregular” rhythm characteristic of AF. This limitation restricts the simulator’s ability to fully replicate the unique hemodynamic patterns observed in AF. We have explicitly noted this limitation in the manuscript as an area for future development. We also emphasize that integrating pneumatic actuators and custom control mechanisms into future iterations of the simulator could provide greater control over rhythm and contraction dynamics, enabling a more accurate representation of AF-specific hemodynamics. We appreciate the reviewer’s insight into this critical area and believe it is a significant avenue for advancing this research.

w) The depiction of left ventricular (LV) pressure in Figure 5B is unrealistic for the presented MCL. The figure illustrates LV pressure during the diastolic phase approaching zero or exhibiting positive values, which is inconsistent for the presented MCL. Given that the circuit connecting the reservoir to the LV consists solely of passive elements, primarily compliance and resistances, and that the reservoir works at atmospheric pressure (approximately zero relative pressure), it is expected that a positive flow would lead to a pressure drop. This drop would result in a negative pressure within the left atrium (LA) model and an even greater negative pressure in the LV.

We apologize for any confusion caused. Based on the suggestions from this Reviewer and others, we have moved these data to the supplementary materials, as the primary focus of our study is on LA dynamics. To enhance transparency, we have clarified the schematic to better detail the MCL setup. The observed LV pressure deviations are likely attributable to limitations of the BDC pump used in the current configuration. Specifically, the pump output may introduce oscillatory components that propagate through the compliance and resistance elements of the MCL, affecting the pressure waveform. This effect is most pronounced during phases of minimal flow, such as diastole, when LV pressure is at its lowest. If the pump lacks sufficient suction capability or prioritizes flow over pressure regulation, it may fail to generate the sub-atmospheric pressures typically observed during LV diastole, resulting in pressures closer to zero or even slightly positive. We now emphasize in the manuscript the potential for future work to incorporate active/contractile LA and synthetic LV integration, which would enable more realistic and physiologically accurate hemodynamic waveforms.

x) Figure 5B presents a non-physiological representation of total pulmonary vein flow ("CO"). The flow is depicted as having a relatively constant positive trend of approximately 4.2 l/min, with a low amplitude of about 0.5 l/min. In contrast, a physiological pulmonary vein flow waveform typically demonstrates a minimum flow around 0 l/min, as illustrated in Figure 1 of the supplementary material. This characteristic is supported by the findings of Fadel, Bahaa M., et al. in their comprehensive review, "Spectral Doppler interrogation of the pulmonary veins for the diagnosis of cardiac disorders," published in the Journal of the American Society of Echocardiography (2021, 34(3): 223-236). The absence of this physiological minimum in the depicted waveform raises concerns regarding the accuracy of the model.

We thank the reviewer for highlighting this important point and acknowledge the non-physiological representation of the waveform amplitude. In response, we have revised the manuscript to report mean LA inflow rather than waveforms and have noted this as a limitation of the current setup. The absence of the negative flow component in the pulmonary vein (PV) waveform arises from the lack of an atrial kick, which is a result of our LA model being passively compliant rather than actively contractile. This limitation prevents the recreation of the characteristic A-wave observed in physiological conditions. We have explicitly highlighted this as an area for future work, emphasizing the potential for incorporating active contractility to address this limitation. Additionally, while it is advantageous for future simulators to achieve this feature, it is worth noting that the loss of the A-wave and a corresponding decrease in waveform amplitude are commonly observed during atrial fibrillation. This observation aligns with evidence in the literature, supporting the use of the current setup for simulating hemodynamic changes characteristic of AF.

y) For accurate validation, the authors should measure the flow at the model outlet to verify the correct imposition of a physiological mitral valve flow, as depicted in Figure 1 of the supplementary material. (see also "Bäck, Sophia, et al. 'Assessment of transmitral and left atrial appendage flow rate from cardiac 4D-CT.' Communications Medicine 3.1 (2023): 22").

We thank the reviewer for this important suggestion and have taken steps to address it by collecting additional data to measure both LA inflow and LA outflow. These measurements are presented in Supplementary Figure 6. We note that the mean LA outflow in our model is lower than the rates reported in the referenced literature; however, this aligns with the expected decline in mitral flow during atrial fibrillation. To further validate our setup, we demonstrate a reduction in LA outflow in Supplementary Figure 4, which recreates key hemodynamic changes characteristic of mitral flow during AF. This comparison strengthens the manuscript and highlights the utility of the simulator in replicating pathological states. We also thank the reviewer for pointing us to the paper by Bäck et al. Our peak mitral flow rates are comparable to those referenced in their study, though we note that the patients in the referenced paper were in normal sinus rhythm, while our simulations replicate AF conditions. Given the well-documented loss of atrial kick and associated reduction in mitral flow during AF, we believe our results align with expectations for this pathological state. In addition, per the reviewer’s suggestion, we have performed comparisons between inflow and outflow, particularly in the context of LAAO and compliance changes. These new data are included in Figure 4, providing further validation of our model. We thank the reviewer for this valuable feedback.

z) To achieve a rigorous validation, the authors should compare the flow at the mitral valve prosthesis (LV inlet) with the flow at the left atrium model outlet. This comparison is critical for assessing the impact of the compliance and resistance components situated between the MV and the LA model.

We thank the reviewer for this suggestion and have conducted the requested comparison. These data are now included in Supplementary Figure 4. As noted in response to comment y) our new data demonstrate the relationship between LA inflow and outflow, especially in the context of LAAO and compliance changes.

aa) The flow waveforms related to the of the Atrial fibrillation test are missing.

We thank the reviewer for this observation. Flow waveforms for the atrial fibrillation test have been included in the supplementary materials (Supplementary Figure 4). The flow waveform and the characteristic reduction in flow observed under simulated AF conditions are now detailed in Supplementary Figure 4. However, we note that the primary objective of this work is to recreate mean inflow and outflow conditions rather than specific waveform replication. This distinction is now clarified in the limitations section and noted as an area for future work and improvement. Additionally, we acknowledge the differences between atrial flutter and AF dynamics in the current setup. Due to the regular nature of the pulsatile pump, the system is better suited to simulate atrial flutter rather than the irregular patterns characteristic of AF. We have highlighted this limitation in the discussion and emphasized the need for further advancements in pump design, such as the integration of a programmable pump, or a contractile model to replace the pump, to achieve more realistic AF dynamics.

bb) The tunability of the MCL was demonstrated solely by displaying the PV flow (Fig. 6A). However, flow modifications at the MV and LA model outlet were not shown.

We apologize for omitting data showing modifications at the LA outflow in the original submission. In response to the Reviewer's comment, we have recreated the setup to measure and display LA outflow, focusing on the impact of varying pump stroke volumes. These additional measurements are now included in Supplementary Figure 6. The results demonstrate the tunability of the system, showing that total LA outflow increases as pump stroke volume is increased at a fixed pump heart rate. These data align with expected trends, further validating the modular and tunable nature of the mock circulatory loop. We appreciate the reviewer’s suggestion, which has strengthened the manuscript by including these additional data.

cc) From Fig. 6A, an increase in SV induces a translation of the waveforms. In contrast, an increase in bpm has different effects: it raises the average, reduces the amplitude, and increases the frequency. I suggest that the authors clarify whether this behavior is solely due to the pump regulation or also influenced by the compliance chambers. In addition, I suggest to explicit why the increase of bpm is related to an increase average flow and a reduction in amplitude, is this dependent on the pump characteristics?

We appreciate the Reviewer’s comment. Following suggestions from this Reviewer and others, we have moved these data to the Supplementary Materials and refocused the analysis on average pressures and inflows, deemphasizing the specific shape of the waveforms. We have acknowledged this as a limitation of the current system. We believe the observed changes are attributable, as the reviewer mentions, to pump characteristics and the pump's inherent limitations in replicating physiological adaptations. In our system, which lacks baroreceptors and adaptive physiological responses, a higher heart rate increases left atrial pressure as the heart has less time to fill, and the ventricle may not empty efficiently, causing blood to back up. Conversely, a larger stroke volume lowers left atrial pressure by providing the heart more time to fill and improving downstream blood flow, effectively reducing pressure in the left atrium. In future work, we aim to incorporate adaptive physiology into the system, such as active feedback mechanisms or more advanced control of pump dynamics, to better replicate physiological responses under varying conditions. Our group has previously implemented adaptive mechanisms in a right-sided MCL^2^.

dd) The system's behavior at 120 bpm is unusual, as it displays the same number of peaks as at 90 bpm (and so same frequency). What could be the cause of this? Additionally, the authors did not present flow data at 160 bpm, which would provide further insights into system performance across frequencies.

We apologize for this error and thank the Reviewer for pointing out the labeling errors in the initial submission. These errors have been corrected, and the data are now included in Supplementary Figure 4.

Regarding system behavior at very high heart rates, such as 160 BPM, we note that reduced filling time significantly limits emptying in the current setup. Additionally, an example waveform at 120 BPM has been included in Supplementary Figure 4 for clarification.

ee) Fig. 6C has a different time scale from Fig. 6A. I suggest unifying the time scale across all graphs in the figure to facilitate comparison.

We thank the Reviewer for this suggestion. We have unified time scales across all graphs to facilitate comparison.

ff) The sentence "Of note, the LAA in our model is not actively contractile (i.e., it does not contract itself, but exhibits passive motion driven by pulsatile flow and its inherent elasticity) and therefore lacks the characteristic a-wave caused by the "atrial kick" or atrial contraction at the end of ventricular diastole just before the mitral valve closes (Figure 5A; Supplementary Figure 1)" represents a discussion point. I recommend relocating this sentence to the discussion section for better context and relevance.

We thank the Reviewer for this suggestion and have relocated this sentence to the discussion, where it provides better context and relevance.

gg) The sentence, "We found that pulse pressure decreases with increasing heart rate (Figure 6C), representative of the pressure waveform observed during atrial fibrillation," lacks clarity. At 120 bpm, the pressure inside the atrium is influenced by a relatively constant flow. Is a constant flow characteristic of the waveform observed during atrial fibrillation?

We thank the Reviewer for highlighting this important question. There are two key points we address here: (1) The regular rhythm at 120BPM in our simulator is closer to atrial flutter rather than true atrial fibrillation, owing to the limitations of the pulsatile pump used. We have acknowledged this limitation in the manuscript under the limitations section. (2) Despite this limitation, we still observe a reduction in flow at higher heart rates in our attempt to simulate AF. This is consistent with the flow reduction characteristic of AF. Supplementary Table 3 summarizes the reduction in flow reported across three sources in the literature. We further demonstrate this reduction in flow amplitude in Supplementary Figure 4. Reduction in flow amplitude is a documented feature of AF dynamics (Chao et al., 2000). Specifically, systolic forward flow is significantly reduced in AF due to the absence of atrial contraction. We have clarified these points in the revised manuscript and included relevant citations to support our findings.

hh) The pressure waveform observed in Figure 7A-C exhibits two peaks during the cardiac cycle, which differs from the waveforms presented in Figures 6C-D. This discrepancy suggests that different compliance values were utilized in this second set of experiments compared to those in Figure 6. What was the rationale behind this choice? The authors should provide the specific values of the parameters used in both experiments to clarify this difference.

We thank the Reviewer for this observation. The Reviewer is correct that different compliance values were used to collect the pressure waveforms in Figure 7A-C compared to those in Figures 6C-D. This discrepancy, which could lead to potential confusion, has been addressed in the revised manuscript. Specifically, we have modified the figures throughout the manuscript for consistency and have removed the data from Figure 7A-C to maintain clarity.

ii) Another point regarding the inconsistencies in the presented results is the observation of a very high amplitude in pressure (approximately 15 mmHg) alongside negative values. This observation suggests that different parameter values for the passive elements were employed in this set of experiments compared to those in Figure 6.

We appreciate the Reviewer’s observation. The presented values are not inconsistent but reflect experimental conditions with modified resistance and compliance settings. By reducing the resistance between the pump and the LA, the LA experienced drastically increased suction from the pump, leading to negative pressures. These data were previously included to demonstrate the platform's adjustability and versatility under different experimental scenarios. However, we recognize that these results may be confusing, and to maintain clarity and consistency, we have removed them from the manuscript. We agree with the Reviewer that these data were collected under different parameter values for the passive elements and represent distinct experimental conditions rather than inconsistencies.

jj) Figure 8 should include the flows at the model outlet.

We appreciate the Reviewer’s suggestion. The initial setup did not include measurements of flow at the model outlet, and these data have now been moved to the Supplementary Materials. Pulmonary vein flow data were originally presented because they showed interesting differences between geometries. The most notable differences were observed in PV pressure and PV flow, and those data were emphasized in the original figure.

kk) The values of the pulmonary vein single flows differ with left atrial appendage geometries. The authors should provide a rationale for these differences to enhance the reader's understanding.

We thank the Reviewer for highlighting this observation. Differences in PV flow behavior likely stem from geometric variability between the patient-derived LAA models, which inherently capture different anatomical features and flow dynamics. Additionally, these differences could be influenced by movement of the model during experimentation or slight adjustments to the flow probes during handling. We have noted in the manuscript that this finding warrants further investigation, as it highlights the need for a deeper exploration of the relationship between LAA anatomy and PV flow behavior. However, this is beyond the scope of the present study. The primary goal of these data was to demonstrate the modularity of the system and its ability to accommodate different anatomies.

ll) I recommend specifying the values of the parameters adopted for the experiments depicted in Figures 8 and 9.

We thank the Reviewer for this suggestion. The relevant parameters have been specified and are now included in the figure captions for Figures 8 and 9 to enhance clarity and ensure reproducibility.

mm) I suggest removing the results related to the Foam Plug, as they do not provide additional information, especially in light of the presence of the real device.

We agree with the Reviewer and have removed these results to streamline the manuscript.

nn) I suggest including the average left atrial pressure in Figure 9 to enhance the understanding of pressure modifications following the implantation.

We appreciate this suggestion; however, our focus is on changes in pulse pressure rather than mean LAP to emphasize the effects of compliance. We worry that including mean LAP could potentially confuse readers, as baseline LAP values were not uniform across all trials. Presenting change in pulse pressure, with each experimental condition serving as its own control, aligns more directly with the primary objectives and findings of this experiment.

oo) The echocardiographic images should be compared with real patient data to assess whether the model allows for realistic visualizations of anatomy and flow dynamics.

We apologize for any confusion regarding the purpose of the imaging in our study. The objective was not to replicate clinical imaging or simulate realistic visualizations of flow dynamics but to utilize imaging modalities as research tools for assessing device performance. Specifically, echocardiography was used to quantify parameters that serve as surrogates for occlusion and device performance, enabling measurable outcomes. This approach involved placing the probe directly on the submerged model, a setup that would not be feasible in clinical scenarios. These imaging methods are intended to demonstrate the simulator’s role as a tool for measurable assessment. We have clarified these objectives and the context of imaging use in the methods, results, and discussion sections.

pp) I recommend that the authors provide more detailed information about the Position, Anchor, Seal, and Size (PASS) parameters and how these were evaluated. It would be beneficial for the authors to report the values of the PASS parameters across different deployment attempts. The ability to quantify these aspects after implantation could serve as a significant strength of the presented simulator.

We thank the reviewer for this valuable suggestion. In response, we have included additional details on the evaluation of the Position, Anchor, Seal, and Size (PASS) parameters in the methods section, following the Directions for Use (DFU) of the WATCHMAN FLX device. These additions emphasize the relevance of PASS parameters for procedural assessment and training purposes. We appreciate the opportunity to further clarify this strength of our simulator in the context of training and education.

**Reviewer #3:** In their manuscript the authors introduce a novel benchtop model for in-vitro hemodynamic analysis of LAA occluders. Their model includes a rigid patient-specific LA geometry and flexible LAAs that can be exchanged in a modular way. The authors show that their setup represents physiological conditions and also showcase its use with several scenarios (occlusion, visualization, training).
It is an interesting topic which is also relevant for the readership of the journal. While the text itself is fairly well written, the study requires more focus and a better presentation and visualization of the results. Substantial changes need to be performed. My general remarks and comments to the individual sections are shown below.

We thank Reviewer #3 for their thoughtful and detailed comments, which have significantly enhanced the clarity and focus of the manuscript. In response to the Reviewer’s feedback, we have made substantial revisions throughout the manuscript to address their concerns and improve its overall quality. Specifically, we have: (1) reduced figures and improved presentation, (2) refined scope and focus, (3) enhanced validation, and (4) revised introduction and discussion. We believe these revisions have resulted in a more focused and cohesive manuscript that effectively communicates the significance and utility of our work. We are grateful to the Reviewer for their constructive feedback and are confident that the manuscript now better reflects the rigor and relevance of our study. Detailed, point-by-point responses to each of the Reviewer’s comments are provided below.

**General remarks:**

1. The number of figures needs to be reduced dramatically. Right now there are 11 figures with several sub-figures and panels resulting in over 30 images. This is way too much. For instance Fig 1-3 could be completely dropped. Maybe keeping one visualization of the final 3d model. The other figures can be either combined or omitted (see comment below).

We appreciate the reviewer’s observation regarding the excessive number of figures in the initial submission. In response, we have significantly reduced the number of figures from 11 to 6 by combining or omitting content as suggested. This revision has streamlined the presentation, enhanced the manuscript’s readability, and strengthened the overall message and impact of the work. We thank the reviewer for encouraging this refinement.

2. All of the figures are dramatically overcrowded. There is a mixture of photographic images, screenshots and plots which are, for whatever reason, all in different colors. Overall design varies as well. E.g. why is Fig. 8 rather blue and punk and Fig. 10 rather orange? Fig 4: Just show a scheme of the setup without the use of 7 different colors. Fig.5-7: Why the different colors and so many curves? Has to be presented in a clearer more compact way. Fig. 8: Is it really necessary? What does it say? Once again, many curves in different intensities of pink/blue. Fig.9 : Only D is relevant. A-C can be omitted. The reader already understood how the 3D printed model looks like. Fig.10: Only A (V) and (VI) is of interest? Everything else can be dropped. Of course, colors should be adjusted and it is not clear why bands are used instead of lines in A (v). Fig. 11: Can be left out completely.

We acknowledge the reviewer’s concerns regarding the overcrowded and inconsistent figures in the initial submission. In response, we have refined all figures by standardizing color schemes for clarity and coherence and have moved extraneous figures to the Supplemental section. We reduced the number of curves and focused on presenting only the most relevant data to ensure clarity. We retained the new Figure 5 to demonstrate the simulator’s compatibility with approved clinical devices and its value in quantitatively assessing degree of occlusion for device testing. This figure reflects collaborative work with Boston Scientific, who recognized the simulator’s value as a research tool. Similarly, we retained the new Figure 6 to highlight the simulator’s utility as a training platform and educational tool. This has received positive feedback from clinicians and industry professionals, particularly for its application in improving deployment techniques and its value as a hands-on learning tool at conferences.

3. Just to emphasize my point here: I think it is really, really important to declutter on this front. Maybe for the authors all the visualizations are clear and logical. But for a reader who has not seen the experiment, it just confusing and "one loses interest" in the study, thus the nice work that the authors have performed will not be acknowledged. Maybe the authors could show their improved figures to other colleagues who are not familiar with the study to see whether they can understand what is communicated in the images.

Again, we apologize for the overwhelming number of figures and graphs in the initial submission and have taken significant steps to address this concern. We have removed unnecessary figures and decluttered the presentation, ensuring that the figures are now more concise and easier to interpret. This refinement has enhanced the overall clarity and digestibility of the manuscript, particularly for readers unfamiliar with the study. We thank the reviewer for recognizing the potential of this work and for pushing us to improve its presentation. We believe these changes make the manuscript stronger and more accessible, significantly enhancing its impact and acknowledgment within the scientific community.

4. This goes hand in hand with my criticism of the figures. The authors also try to cover too much in their study. It includes test bench creation, proving that it is physiological, comparing devices (a bit), visualization and imaging (a bit) and even training (a bit). Everything is just a bit here and, provocatively said, after reading the manuscript I am left with the feeling of "Yes, it seems to work. But I am also not really and to what degree does it really work." I am providing specific suggestions later on.

We thank the reviewer for this criticism and have carefully revised the manuscript to address these concerns. We have placed greater emphasis on the validation of our simulator to demonstrate its accuracy and functionality, providing clearer evidence that it works as intended. In doing so, we have reduced the focus on visualization, imaging, and other applications to avoid overstretching the study. While we maintain that the simulator is a versatile and multifunctional platform, the revised manuscript prioritizes validating and recreating physiological hemodynamic data. This strengthened emphasis ensures that the utility and accuracy of the simulator are convincingly established before showcasing its broader applications.

**Introduction:**

5. Well written, but it suffers from the same points as mentioned above. Please - focus, focus, focus! 70 studies are cited which is way too much for an original research article. Further on, the authors talk a lot about computational models and also ex-vivo setups. But why not about in-vitro setups and their limitations and just leave out the other parts? Strangely, the limitations of ex-vivo models are discussed with many details (which would imply that the authors are aiming to address those), but as the work is on in-vitro setups, why should we even bother with such detailed descriptions of ex-vivo setups? Just mentioning that, apart from in-vitro, one could also use in-silico or ex-vivo (and citing 2-3 studies) is more than enough. Then, the authors need to clearly define the scientific gap (!!!) of the in-vitro studies that they will address and hopefully close in their work.

We thank the reviewer for this constructive comment. In response, we have revised the introduction to make it more focused and streamlined, ensuring that our work is situated within a more relevant context. We have significantly reduced the discussion on ex-vivo setups and instead placed greater emphasis on in-vitro setups and their limitations. Furthermore, we have reduced the number of citations and concentrated on directly relevant references. Most importantly, we have worked to clearly define the scientific gap in in-vitro studies that our work addresses and how it advances the field. We appreciate the reviewer’s critique and believe that these revisions have resulted in a stronger, more focused introduction that clearly conveys the purpose and significance of our study.

**Methods:**

6. This is the clearest section with brief and specific information that is well-structured. My only remark is the reason why the authors are stressing so much the 100 CT scans that they have looked at. It is not the case that they have used kind of a "population-averaged LA geometry" or something that would justify the mentioning of these numbers. In the end, they selected 1 patient whose LA was segmented and 4 other LAAs were taken (probably from at least three other patients). However, information on age/gender of the patient would be helpful. Is this a physiological case or an extreme case that was selected and what was the reasoning to select the specific geometry? Same goes for the LAA. Any selection criteria for selection?

We thank the reviewer for their comments on the clarity of the methods section. We selected a single patient geometry from a library of 100 anonymized scans. To address the reviewer’s concerns, we have updated the methods to include more details about the selection process and criteria. Specifically, we selected a geometry from an AF patient that met predefined criteria, such as left atrial dilation and a long-axis dimension exceeding 32 mm. Unfortunately, due to the anonymized nature of the database, we do not have access to the age or gender of the selected patient. We apologize for any confusion and believe the additional details clarify the rationale and context for our selection.

7. As the LA is also flexible in reality and its movement (physiological/pathological) will be probably very critical, especially during AF. What is the rationale for using a rigid model? Why not having everything flexible?

The reviewer raises an excellent point regarding the flexibility of the LA. In this study, we opted to use a rigid LA model to facilitate the modular exchange of multiple LAA geometries. Designing attachment features for soft geometries proved challenging and impractical for this initial iteration. While it is feasible to 3D print the entire geometry as flexible, this approach would require reprinting for each individual patient case, significantly increasing resource and time demands. Using a single rigid LA model with multiple interchangeable LAA geometries allows for more efficient experimentation. We have acknowledged this limitation in the discussion and identified it as an area for future work, as we agree that incorporating flexibility in the LA would enhance the physiological relevance of the model.

8. Concerning the experimental setup in Fig.4. By looking at in detail, I have some questions regarding its structure. What is the reason of having two fluid compartments with a resistance downstream of the LA and upstream of the MV? Normally one would expect the mitral valve being directly "after" the LA. Actually, in this case, a photograph of the entire experimental setup would help tremendously to understand the setup.

The reviewer raises an important point regarding the structure of the experimental setup. We have now included a photograph of the complete setup to improve clarity and understanding. We acknowledge that, physiologically, the mitral valve would typically be positioned directly after the LA. However, in this model, the inclusion of two fluid compartments with a resistance element downstream of the LA and upstream of the MV was necessary to mitigate the suction effect caused by the non-physiological pumping mechanism. This design choice reflects a limitation of not incorporating an active LV. The compliance and resistance elements serve to dampen non-physiological suction from the pump, ensuring stable flow dynamics. We have clarified this limitation in the methods section and discussed it in the limitations of the current setup. Future iterations could include an active LV, enabling a more physiological pumping mechanism and flow dynamics.

**Results:**

9. In my opinion, the first three paragraphs are Methods and not results. Especially, as several parts are just repeating of methods (e.g. the open-source database was used to segment CT)… The first "real" result starts with Figure 5 where you show that your experimental setup produces realistic values.

We thank the reviewer for this comment. We have refined the methods and results sections to reduce redundancy and enhance clarity. Additionally, we combined figures to streamline the presentation of data. However, we consider the first three paragraphs to be part of the results because they reflect the process of creating the simulator, which is one of the primary objectives of this work. The simulator is presented as a tool and model that can be used for future research and for addressing specific research questions. The segmentation, modification, 3D printing, and silicone casting are integral steps in the development of this model, and we believe these are key outcomes that belong in the results section.

10. Figure 5 and its description. Please, think of better ways to prove that your setup produces realistic values. Fig.5A can be omitted. B looks nice, but why are these values so "clean"? Filtering was performed. How? What were the beat to beat variations and the differences in values between LAA1 and LAA2-4? Why is only LAA1 shown? Where were the physiological values taken from?

We thank the Reviewer for these comments. In response, we have created a new Figure 3 to present a clearer and more comprehensive representation of the validation process. Figure 5 has been removed, with some elements moved to the supplementary materials for reference. Regarding the data originally presented in Figure 5, no filtering was applied, and beat-to-beat variations were minimal due to the controlled nature of the experimental setup. Differences between various LAA geometries are now included in Supplementary Figure 2, while detailed hemodynamic comparisons across geometries are presented in Supplementary Figure 6. Healthy reference data were sourced from UpToDate, and AF-related data were obtained from a clinical cohort at Mayo Clinic, supplemented with additional values from the literature. These references are all summarized in Supplementary Tables 2-6. We believe these changes address the reviewer’s comments and improve the clarity and presentation of our validation data.

11. Fig. 5C: Why comparing the inflows into the atrium to a total CO? Is this a literature value? Normally cardiac output (if it is the flow from left ventricle to aorta) looks very different…

We thank the Reviewer for pointing out this issue and apologize for any confusion. In response, Figure 5 has been removed, with some elements relocated to the supplementary materials for reference. To improve clarity, we have renamed flow values to “LA inflow” and “LA outflow” where appropriate. We acknowledge that using “total CO” was not the most precise terminology and have revised this to avoid misinterpretation. Additionally, we agree with the Reviewer that the waveform in question differs from physiological cardiac output waveforms. To address this, we have deemphasized specific waveform characteristics and instead focused on mean values, which are more representative of the study’s objectives. We believe these changes help to clarify our findings and address the Reviewer’s concerns.

12. Fig 5D: Why are there so many curves? You should just compare the LAA pressure (that was measured through a catheter???) with the LA healthy and LA AF values. So basically, we see that your setup can mimic pressures for AF patients but is higher than healthy values. Once again, where did you take these values from? How did it look like for the other LAA geometries?

We thank the Reviewer for highlighting this issue and apologize for the confusion caused by the overwhelming number of curves originally presented. In response, we have removed Figure 5 and relocated relevant elements to the supplementary materials for reference. Pressure within the LAA was measured using a catheter, and we have updated the methods section to clarify this measurement process.

To address the Reviewer’s concerns, we have included a new Figure 3 that focuses on validation and demonstrates that our setup can accurately reproduce both healthy and pathological pressures. The validation data were collected using different LAA geometries, with LAA1 representing a healthy reference and LAA2-4 reflecting AF patient geometries. Specific differences in hemodynamics across these geometries, under identical pump and flow loop conditions, are now detailed in Supplementary Figure 8. We appreciate the Reviewer’s comments and believe the revised presentation resolves these concerns, while providing a more concise and informative depiction of our findings.

13. You already see that just the first aim of your study - "validating" the model against (literature? physiological/pathological) values requires much more work. The results presented are just not enough to really prove that your benchtop setup works. I strongly, strongly, strongly recommend to take a step back, reduce the amount of goals/variety of investigations but do these investigations (e.g. the proof that your in-vitro setup behaves realistically) in a more thorough way.

We thank the Reviewer for this suggestion. We fully agree that validation is a critical component of this study and have taken significant steps to address this concern. In response, we have placed greater emphasis on validation throughout the manuscript. We have shifted focus away from the morphology of waveforms to instead highlight target mean values. Additionally, we performed further flow validation and ensured reproducibility of results. We have also refined the overall goals of the manuscript to prioritize validation of the simulator and its use for quantifying metrics that are directly relevant to device testing and development. The manuscript now reflects these revised goals, emphasizing the simulator as a tool for producing measurable and quantifiable surrogates for device evaluation and research. We thank the Reviewer for this constructive feedback, which has strengthened the manuscript significantly. We believe the revised study now demonstrates that the benchtop setup behaves realistically and can be used reliably to assess device performance with quantifiable measures of success.

14. In my opinion the content of Figures 6 and 7 (and the presentation of the results) is not so relevant in the context of the study. Yes, changing the frequency of the pump will change the cardiac output and using a different material for the LAA will change its stiffness. But these are secondary results, you first would really need to show that your setup is working, creates realistic and reproducible (!!!) pressures and flows. As of now, the results do not support this statement.

We thank the Reviewer for this valuable feedback. In response, Figures 6 and 7 have been moved to the supplementary materials to allow the main manuscript to focus more directly on demonstrating realistic and reproducible pressures and flows. While these figures initially aimed to highlight the system's tunability and adaptability, we agree that the primary focus should be on validating the setup's physiological relevance. The system’s tunability remains a significant advantage, enabling customization for diverse research goals, particularly in device design and evaluation. However, to better align with the Reviewer’s recommendation, we have refined the presentation to prioritize demonstrating the system's reliability and reproducibility, noting that calibration and adjustment are critical for achieving experimental alignment with specific objectives.

15. Same goes for the Figures and the descriptions of 8-11. Too superficial and I am very skeptical of the value that those investigations provide (apart from showing some graphs and photos).

We thank the Reviewer for their feedback and understand the concerns regarding Figures 8–11. These figures were included to demonstrate the simulator's utility for both clinical and industry purposes, as highlighted through collaborative efforts with Boston Scientific (BSC) and feedback from other stakeholders. Specifically, Figures 10 and 11 showcase applications that were well-received by BSC, who have recognized the simulator’s value in device evaluation and development. This work has also garnered positive feedback from clinicians and industry representatives, particularly for its role in improving deployment techniques and its value as an educational tool. BSC is actively utilizing the simulator in developing new devices, emphasizing its ability to provide measurable, quantifiable metrics for assessing device performance and functionality. Furthermore, the simulator has proven beneficial in educational settings, allowing clinicians to engage in hands-on training and deployment practice.

**Discussion:**

16. I would need to re-read it when focus and content of the manuscript has been adjusted. But as a general comment, the Discussion is basically repeating the observations made. It has to:
    a. repeat the aim
    b. summarize the results
    c. put them in context w.r.t. to the state of the art and the overall problem
    d. discuss limitations and possibilities
    e. provide the "take home message" and "bigger picture" of the work.

We thank the Reviewer for outlining their expectations for the discussion. In response, we have rewritten the discussion section to align with the suggested structure, incorporating all changes made to the manuscript and reflecting the new areas of focus.

**Reviewer #4:** Dear Authors of the Manuscript, Congratulations on the great work and the implementation of the setup to train, test and observe LAA occluders deployment. There is an unmet need for such a simulator - the setup might be particularly useful for industry representatives during conferences and hands-on sessions to train physicians and to showcase devices. Currently they use mentice workstations and very crude plastic setups (attended such an event this month). The manuscript is written well, in really plain and easy-to-understand language. Here's just a couple of potential improvements to enhance the reception of the article:

We sincerely thank Reviewer #4 for their positive and encouraging feedback on our work. We deeply appreciate the thoughtful suggestions and insightful comments, which have helped us refine and enhance our manuscript. The recognition of the utility and relevance of our simulator, particularly for industry representatives and educational applications, is highly motivating. Detailed, point-by-point responses to each of the Reviewer’s comments are provided below.

1. The abstract could be more brief, now it is too wordy.

We thank the Reviewer for this suggestion. We appreciate the push to improve clarity and conciseness. In response, we have revised the abstract to be briefer while retaining all critical details of the study.

2. Introduction: instead of left circumflex artery, better use circumflex artery (Cx)

We thank the Reviewer for this observation. We have corrected the terminology.

3. The same size and morphology LAAs may accommodate Watchmans of different diameters (according to the manufacturer's chart), but the radial expansion will be different (compression % as the industry reps call it, the reps may share the charts they have with occluder diameter/compression %/sizes of LAA as measured by TEE/ICE). That is why it is important to test occluders of different sizes: 20mm, 24mm, 27mm, 31mm, 35mm

We thank the Reviewer for this excellent insight. We agree that testing occluders of various sizes is a significant potential advantage of our simulator. In response, we have noted in the manuscript that the simulator supports deployment of multiple device sizes within the same geometry. We have also included a discussion of using compression percentages as a valuable metric for device testing, which aligns with current industry practices. Additionally, we have proposed the integration of force sensors in future iterations to assess device compression more comprehensively. These updates are reflected in both the results and the discussion sections.

4. The correct term is Wiggers diagram, not "Wigger" Figure 5A, as the surname is Wiggers

We thank the Reviewer for noting this error. We have corrected the term to "Wiggers” throughout the manuscript.

5. Please, mention that this setup can be used by industry for conference setups

We thank the Reviewer for this excellent point. We agree that the simulator has strong potential for use in industry conference setups to train physicians and showcase devices. In response, we have updated the discussion to include this additional advantage of the simulator.

6. Please, mention the alternative of procedural simulators such as educational modules by Mentice, or other manufacturers

We thank the Reviewer for sharing this information about procedural simulators. These tools, such as those developed by Mentice, are indeed valuable for clinical education. We have updated the Introduction to include mention of VR procedural simulators as important educational tools and have referenced Mentice to provide context. We are grateful for this suggestion, which helps position our simulator alongside other valuable educational resources.

**References**

1.	Nicol, E.D. (2020). Using CT left atrial appendage imaging to identify patients at higher risk of stroke. J Cardiovasc Comput Tomogr *14*, 34–35. https://doi.org/10.1016/j.jcct.2019.05.013.

2.	Singh, M., Roubertie, F., Ozturk, C., Borchiellini, P., Rames, A., Bonnemain, J., Gollob, S.D., Wang, S.X., Naulin, J., El Hamrani, D., et al. (2024). Hemodynamic evaluation of biomaterial-based surgery for Tetralogy of Fallot using a biorobotic heart, in silico, and ovine models. Sci Transl Med *16*. https://doi.org/10.1126/scitranslmed.adk2936.
